# Comparative meta-analysis of transcriptomic studies in spinal muscular atrophy: comparison between tissues and mouse models

**DOI:** 10.1186/s12920-024-02040-0

**Published:** 2024-11-12

**Authors:** Shamini Hemandhar Kumar, Katharina Brandt, Peter Claus, Klaus Jung

**Affiliations:** 1 Institute for Animal Genomics, University of Veterinary Medicine, Foundation, Buenteweg 17P, Hannover, D-30539 Germany; 2 SMATHERIA gGmbH – Non-Profit Biomedical Research Institute, Hannover, Germany; 3 Center for Systems Neuroscience (ZSN), University of Veterinary Medicine, Foundation, Hannover, Germany

**Keywords:** Differential expression analysis, Meta-analysis, Network reconstruction, Spinal muscular atrophy, Transcriptomics, WGCNA

## Abstract

**Background:**

Spinal Muscular Atrophy (SMA), a neuromuscular disorder that leads to weakness in the muscles due to degeneration of motor neurons. Mutations in the survival motor neuron 1 (*SMN1*) gene leads to the deficiency of SMN protein that causes SMA. The molecular alterations associated with SMA extends across the transcriptome and proteome. Although several studies have examined the transcriptomic profile of SMA, the difference in experimental settings across these studies highlight the need for a comparative meta-analysis to better understand these differences.

**Methods and data:**

We conducted a systematic comparative meta-analysis of publicly available gene expression data from six selected studies to elucidate variations in the transcriptomic landscape across different experimental conditions, including tissue types and mouse models. We used both microarray and RNA-seq datasets, retrieved from Gene Expression Omnibus (GEO) and ArrayExpress (AE). Methods included normalization, differential expression analysis, gene-set enrichment analysis (GSEA), network reconstruction and co-expression analysis.

**Results:**

Differential expression analysis revealed varying numbers of differentially expressed genes ranging between zero and 1,655 across the selected studies. Notably, the Metallothionein gene Mt2 was common in several of the eight comparisons. This highlights its role in oxidative stress and detoxification. Additionally, genes such as Hspb1, St14 and Sult1a1 were among the top ten differentially expressed genes in more than one comparison. The Snrpa1 gene, involved in pre-mRNA splicing, was upregulated in the spinal cord and has a strong correlation with other differentially expressed genes from other comparisons in our network reconstruction analysis. Gene-set enrichment analysis identified significant GO terms such as contractile fibers and myosin complexes in more than one comparison which highlights its significant role in SMA.

**Conclusions:**

Our comparative meta-analysis identified only few genes and pathways that were consistently dysregulated in SMA across different tissues and experimental settings. Conversely, many genes and pathways appeared to play a tissue-specific role in SMA. In comparison with the original studies, reproducibility was rather weak.

**Supplementary Information:**

The online version contains supplementary material available at 10.1186/s12920-024-02040-0.

## Introduction

Spinal muscular atrophy (SMA) is a genetic neuromuscular disorder caused by the degeneration of motor neurons in the spinal cord and brain stem leading to muscle weakness [24, 33]. The survival motor neuron 1 (*SMN1*) gene is either deleted or shows point mutations, which in turn leads to the deficiency of the SMN protein. As a consequence, the decrease of SMN causes to transcriptomic and proteomic alterations. Currently, five types of SMA are known, where Type 0 is the most severe and rare form, developing already prenatally (Mercury et al., 2022). Therapies that have been developed so far target the SMN protein, e.g. by replacing SMN1.

A number of studies has been performed to study and investigate the transcriptome under SMA. These studies focus on the transcriptome in different organs and under different experimental settings which are performed at different time points. While individual transcriptomic studies provide insights under specific experimental settings, the aim of this work is to systematically re-analyse publicly available gene expression data of a selection of these studies to better understand the differences in the transcriptome under different settings such as different organs and mouse models. Moreover, the scientific evidence of individual studies is usually limited [6] [29] and only the synthesis of multiple research outcomes can provide a more conclusive picture. The selected studies in our comparative analysis involve both microarray and RNAseq study types [7]. Data of high-throughput gene expression, measured by DNA microarrays or RNAseq, can be retrieved from public databases such as Gene Expression Omnibus (GEO, https://www.ncbi.nlm.nih.gov/geo/) [1, 18] or BioStudies-ArrayExpress (AE, https://www.ebi.ac.uk/biostudies/arrayexpress) [49]. Raw public gene expression data opens up to conduct classical meta-analysis, which means to combine results from individual studies in the form of fold change or *p*-value combination [38, 46]. Alternatively, raw data can be merged, batch-effect corrected [32, 60] and analysed as a whole [57].

While classical meta-analysis aims to combine studies of the same setting in order to increase statistical power [53] and thereby scientific evidence and reproducibility [6] [52], we focus here on studies with different settings in a comparative way. Comparative meta-analysis allows to study the differences in the transcriptomic changes in different organs, species or in general under different experimental settings [48, 54]. The advantage of doing a comparative meta-analysis with raw data – in contrast to a literature review – is that all steps of analyses on the individual studies can be harmonized. In this study, our data analysis includes standard analyses such as normalization, differential expression analysis, gene-set enrichment analysis (GSEA). In addition to the method spectrum of the original publications, we included network reconstruction and co-expression analysis. While data were analysed with different approaches in the original publications with a different aim of study, the same methods for data analysis can be used in a comparative meta-analysis. Additionally, original publications often do not provide the full set of results such as gene lists with fold changes.

In this study, we detail the process of selecting studies and the data sets included as well as our approach for comparative analysis. Thereafter, the results of differential expression analysis, gene-set based enrichment analysis, network reconstruction and co-expression analysis are presented. We conclude with a discussion of the computational approach and their corresponding biological findings.

## Methods and data

In the following we describe the selection of relevant datasets from the GEO and AE databases, the methods for data preparation and normalization, differential expression analysis and GSEA, as well as for network reconstruction and co-expression analysis. All data analyses were performed in the R programming environment ([43], www.r-project.org, V4.3.2).

### Database search and data selection

A systematic search was performed in Gene Expression Omnibus (GEO) and Array Express (AE) using the search term ‘Spinal Muscular Atrophy’ following the ‘Preferred Reporting Items for Systematic Reviews and Meta-Analyses’ (PRISMA) guidelines [42]. A total number of 113 hits from GEO and 111 hits from AE were retrieved on 09 August 2023. Studies that were not related to ‘Mus musculus’ were excluded. Next, studies from GEO that were also in AE were removed. Furthermore, studies that were not SMA and not a series were excluded. Lastly, in order to meet the selection criteria for meta-analysis, series that still had other diseases, no control group and no proper data were excluded. Finally, 6 studies were included for meta-analysis (Supplementary Data S1 SF1). These studies include three microarray and three RNA seq study types, where cells and tissues included are skeletal muscle, brain, motor neurons, spinal cord and embryonic stem cell- derived motor neurons. All GSE series matrix files for microarray data were downloaded into the R programming environment using the GEOquery R-package from Bioconductor (www.bioconductor.org, V3.2). Raw FASTQ-files for RNA-seq data were downloaded using the SRAToolkit [34]. A summary of the six selected studies is given in Table 1.

**Table 1:** Detailed information on the six selected studies including their GEO or Array Express accession ID and their corresponding Pubmed references

GEO or AE ID	Sequencing or microarray platform	Study type* MA/RNAseq	Per group sample sizes (control, diseased)	Tissue	Mouse model	References
GSE207890	Agilent-028005 SurePrint G3 Mouse GE 8 × 60K	MA	8 Control, 12 Smn1deltaSKM, 7 Smn1deltaSKM + AFS	Skeletal muscle	Smn1 knockout 3 months-old, Smn1 knockout treated with amniotic fluid stem	[10]
GSE154106	Illumina HiSeq 2500	RNA Seq	4, 4	Brain	Severe Taiwanese SMA mouse model	[31]
GSE102204	Illumina HiSeq 2000	RNA Seq	10, 10	Brain	Early-symptomatic and late-symptomatic	[3]
GSE56284	Illumina HiSeq 2500	RNA Seq	3, 3	Embryonic stem cell-derived motor neurons	Mouse embryonic stem cell model	[37]
GSE10224	Affymetrix Mouse Expression 430A Array	MA	3 (Control), 3 (SMA), 3 (Transplanted SMA)	Motor neurons of the spinal cord	Transgenic SMA and transplanted mice	[13]
E-MEXP-2428	Affymetrix GeneChip Mouse Exon 1.0 ST Array	MA	8 (normal), 7 (SMA)	Whole spinal cord	Severe mice model	[41]

^*^MA Microarray, *RNA Seq* RNA Sequencing, *GEO* Gene Expression Omnibus, *AE* Array Express

### Data preparation and differential analysis

RNA-seq data (GSE154106, GSE102204 and GSE56284) were obtained from Sequence Read Archive (SRA). SRA sample files were downloaded using the prefetch command from SRAToolKit [34]. The software FastQC [56] was used for quality control, CutAdapt [39] was used for trimming and removing the adapters, the STAR tool [16] for mapping to the mm39 genome, and duplicates were removed using the picard tool (http://broadinstitute.github.io/picard/) and finally feature counts [35] was used to count the reads. Differential expression analysis was performed for these count data using the DESeq2 R-package [36]. For primary identification of differentially expressed genes, we used a cut-off of 5% for FDR-adjusted *p*-values according to the method of Benjamini and Hochberg [2].

To select genes for the intersection analysis between the different studies, we used two additional selection criteria. First, a combined criterion requiring an FDR-adjusted *p*-value < 0.05 and an absolute logFC > 2, in order to avoid too many false positives. Second, in order not to miss relevant genes with a weak effect but having maybe biological importance we also studied the set of top 500 genes in each comparison, ranked by their raw *p*-values*.*

Microarray data of the studies GSE207890 and E-MEXP-2428 were not yet log-transformed, so we performed this step prior to normalization. However, the dataset GSE10224 was already publicly provided with the log-transformed version. All microarray datasets were normalized using quantile normalization [4] and differential expression analysis was performed using the R-package limma [47].

Differential expression analyses were done between a control group and a diseased group using the limma-package. For those studies that had more than two groups (GSE207890 and GSE10224), multiple differential analyses were done. In particular, for dataset GSE207890, the comparisons include Smn1 knockout treated with amniotic fluid stem (Smn1deltaSKM + AFS) versus Ctrl (Control group) and Smn1 knockout (Smn1deltaSKM) versus Ctrl. Furthermore, for dataset GSE10224, the comparisons included SMA versus Ctrl and transplanted SMA (transSMA) versus Ctrl. Thus, a total of eight different group comparisons were performed.

### Gene-set enrichment analysis

GSEA for Gene Ontology (GO) terms: biological processes (BP), molecular functions (MF) and, cellular components (CC) was performed subsequent to all eight differential expression analyses using the clusterProfiler R-package [58]. We chose GSEA instead of over representation because it is only based on the ranking of genes and not on a particular set of differentially expressed genes. Due to the different study settings, having such sets available can be a problem. In addition to the GO enrichment analysis, enrichment analysis for Kyoto Encyclopedia of Genes and Genomes (KEGG) and Reactome pathways were performed using clusterProfiler, too. To analyse the robustness of the GSEA results, we used the bootGSEA R-package [23] to understand and interpret the robustness of GO terms and pathways. This approach repeats GSEA multiple times with subsets of the gene sets based on bootstrap approach to see whether individual genes have an effect on the outcome of GSEA. The bootstrap based analyses resulted in original and bootstrap ranks for each GO terms and pathways. The resulting tables, ordered by bootstrap ranks, show the difference in ranks between original and bootstrap analyses, thereby providing robust assessment of GSEA.

The biomaRt R-package [17] was used in order to get the GO annotations from the Gene Ontology database (https://geneontology.org/).

### Network reconstruction

To better understand the interplay between the most significant genes across all comparisons in each study group, the union of top5 differentially expressed genes from all 8 comparisons was built. Thus, a reconstructed network for a particular study group involved also genes that might not be relevant in this tissue but in another tissue under SMA. We limited the input to the top5 from each comparison in order to obtain too large and too hard to interpret networks.

Networks were constructed for these genes with their count matrix for RNA sequencing studies and expression levels for microarray studies using the minet package [40] in R which helps in inferring mutual information networks to understand gene interactions. These networks were then visualized using the igraph R package [14], providing a comprehensive view of the interactions and relationships between the key genes identified.

### Co-expression network analysis

To further understand the role of genes with similar expression, Weighted Gene Co-expression Network Analysis (WGCNA) was used to analyse and identify co-expressed genes within SMA and wild type groups individually. The microarray expression data were normalized individually for SMA and wildtype groups using quantile normalization from limma R-package. RNA seq data on the other hand, normalization was done using the normalize function from broman Rpackage for SMA and wild type groups separately.

WGCNA R package [30] was used to construct a co-expression network based on pairwise correlations between genes using the topological overlap matrix (TOM), where a soft-threshold power was determined using the scale independence criteria to approximate a scale-free topology. Furthermore, minimum cluster size parameter was adjusted between 100 and 800 in order to obtain biologically relevant modules for study groups (Supplementary Data S2) and cutHeight was set to 0.98 for all groups. Genes were clustered based on dissimilarity measure (1-TOM).

Top Hub genes were identified for modules related to neuromuscular disease using the R function “chooseTopHubInEachModule” in WGCNA R package and studied for their biological significance in SMA. Furthermore, common genes among the modules between SMA and wild type have been analysed to explore potential gene signatures with respect to SMA.

## Results

Our approach towards comparative meta-analysis revealed significant overlaps in differentially expressed genes and enriched gene-sets across the selected studies. Furthermore, networks were constructed from the resulting top 5 differentially expressed genes (DEGs), providing information into the interconnections and potential regulatory relationships among these genes.

### Differential expression analysis

The eight differential expression analyses were performed between the control and diseased groups. The numbers of selected genes, according to different criteria are listed in Table ST1 of Supplementary Data S1. After FDR-adjustment, there were three comparisons with a very large number (several thousand) of significant genes, two comparisons with a moderate number (several hundred) of significant genes, and three data sets with zero or only one significant genes. The largest effects (i.e., the largest portion of differentially expressed genes) by SMA on the transcriptome were seen in the studies on skeletal muscle (GSE207890) and embryonic stem cell-derived motor neurons (GSE56284). Moderate effects were also seen in the study on mouse brain tissues (GSE154106 and GSE102204). The analyses based on motor neurons of the spinal cord (GSE10224) and the whole spinal cord (E-MEXP-2428) yielded no or only one significant gene after *p*-values adjustment.

**Fig. 1:**
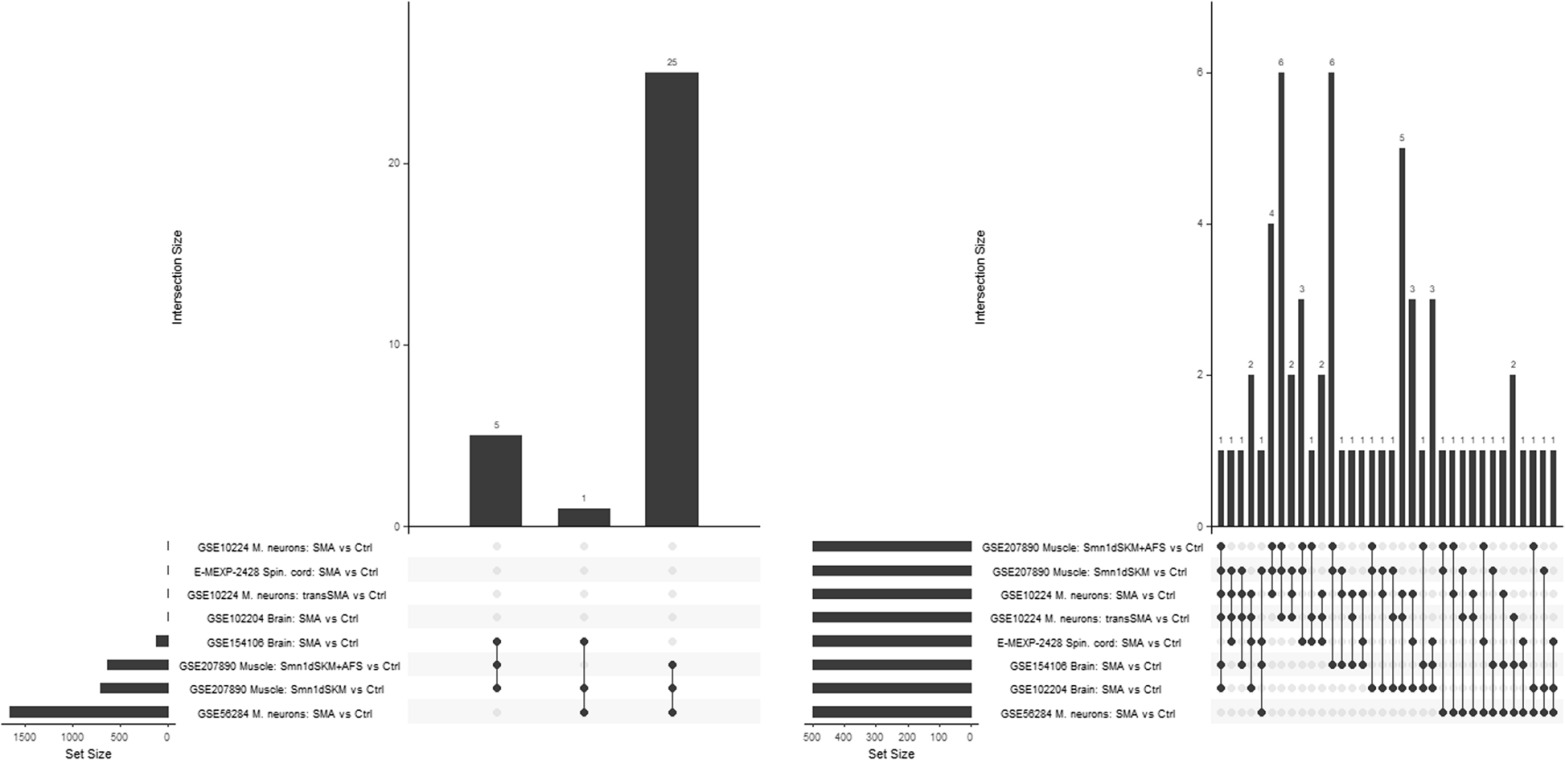
Upset plots [12] of common DEGs among all the group comparisons based on either the selection criterion with an FDR-adjusted *p*-values < 0.05 and an absolute logFC > 2 (left) or on the criterion of being among the top500 (right). Only intersections among at least three comparisons are shown

Due to these different sizes of effects, and because the overall number of genes in each experiment was quite different, we used two different criteria for intersection analysis. With the first – rather strong – criterion (adjusted *p*-value < 0.05 and absolute logFC > 2), we identified 31 genes which occurred in at least 3 out of 8 comparisons (Fig. 1, left). With the second criterion (gene among the top500), 61 genes occurring in 3 out of 8 comparisons, were selected (Fig. 1, right). The genes of these two sets are reported in Table ST2 of Supplementary Data S1. The overlap of both sets were the four genes Hbegf, Mt2, Sim1, and Sox9. While Mt2 was also among the top10 in the comparison ‘GSE10224: SMA versus Ctrl’, none of the three other genes was observed among the top10 from each comparison (Table 2). Among the top10 lists, only St14 and Sult1a1 occurred twice. Apart from these two genes, the top10 lists were disjunct.

**Table 2:** Top 10 differentially expressed genes from the 8 group comparisons with raw and FDR-adjusted *p*-values as well as the log fold change

GSE207890: Smn1δSKM + AFS versus Ctrl / Skeletal muscle				GSE207890: Smn1δSKM versus Ctrl / Skeletal muscle			
Gene.ID	*p*-value	*p*FDR	logFC	Gene.ID	*p*-value	*p*FDR	logFC
Apoa1	5E-15	3E-10	-2.88	Mbd5	2E-14	6E-10	2.59
Havcr1	2E-14	3E-10	-2.78	P4ha1	4E-14	6E-10	2.41
1700057K13Rik	3E-14	4E-10	-2.75	Smek2	4E-14	6E-10	2.29
Gm5567	4E-14	5E-10	-2.74	Rrad	7E-14	8E-10	6.51
Sh3bgrl2	1E-13	8E-10	-2.86	Fam134b	3E-13	3E-09	2.96
Gm13807	3E-13	1E-09	-2.36	Chac2	5E-13	4E-09	2.51
Ribc2	3E-13	1E-09	-2.40	H60b	1E-12	9E-09	-2.66
Lef1	4E-13	1E-09	-2.62	Sap30	2E-12	1E-08	3.96
Myl7	4E-13	2E-09	-2.78	Egln2	2E-12	1E-08	4.69
Mical2	6E-13	2E-09	-2.62	Zcchc7	2E-12	1E-08	2.54
**GSE10224: SMA versus Ctrl / Motor neurons**				**GSE10224: transSMA versus Ctrl / Motor neurons**			
Gene.ID	*p*-value	*p*FDR	logFC	Gene.ID	*p*-value	*p*FDR	logFC
Hspb1	2E-05	0.45	3.36	1700067P10Rik	2E-06	0.04	3.72
**St14**	7E-05	0.45	2.08	Nr0b1	6E-06	0.07	-2.80
Slco1b2	9E-05	0.45	3.03	Aste1	1E-05	0.08	-2.78
Igk-V1	1E-04	0.45	-2.51	Xist	2E-05	0.08	4.28
LOC100043315	1E-04	0.45	2.67	Myh1	2E-05	0.08	-3.00
**Sult1a1**	1E-04	0.45	2.34	4921530L21Rik	4E-05	0.10	-2.57
Cdkn1a	1E-04	0.45	2.70	Cops5	4E-05	0.10	3.00
Pcdhb14	2E-04	0.47	-3.17	Epha7	5E-05	0.10	-2.36
Hspb1	3E-04	0.48	2.07	**St14**	6E-05	0.10	2.09
Mt2	3E-04	0.48	1.84	Rd3	7E-05	0.20	-2.23
**E-MEXP-2428: SMA versus Ctrl / Spinal cord**				**GSE154106: SMA versus Ctrl / Brain**			
Gene.ID	*p*-value	*p*FDR	logFC	Gene.ID	*p*-value	*p*FDR	logFC
Smn1	5E-06	0.08	-1.57	Eola1	0.0193	4E-28	2.22
F2rl2	8E-05	0.70	0.57	1110059G10Rik	0.0040	4E-28	3.49
Snrpa1	3E-04	1.00	0.56	Cfap90	0.0081	4E-26	2.38
Chodl	5E-04	1.00	-0.43	Smim30	0.0031	3E-25	2.35
Eda2r	8E-04	1.00	0.45	Norad	0.0053	3E-25	-2.15
Mccc2	1E-03	1.00	-0.31	4921524J17Rik	0.0044	7E-23	2.92
Zc3h6	1E-03	1.00	0.22	9430037G07Rik	0.0000	2E-22	-4.44
Olfr103	1E-03	1.00	0.22	Abca7	0.0045	1E-19	-2.09
Ppt1	1E-03	1.00	0.53	Abt1	0.0406	3E-19	2.04
Ifna2	2E-03	0.20	0.44	Snord43	0.0000	1E-18	-3.36
**GSE102204: SMA versus Ctrl / Brain**				**GSE56284: SMA versus Ctrl / Motor neurons**			
Gene.ID	*p*-value	*p*FDR	logFC	Gene.ID	*p*-value	*p*FDR	logFC
Ccl21a	1.2E-57	2E-53	-2.88	Gm30414	0.0473	< 1E-256	3.47
Aplnr	1.2E-45	1E-41	-1.54	Cdh7	0.0009	< 1E-256	-2.37
Rgs5	1.7E-33	1E-29	-0.62	9330185C12Rik	0.0000	1E-237	3.23
Pla2g3	3.8E-32	2E-28	0.79	Asic4	0.0000	5E-234	-4.37
Tnfsf10	2.3E-29	9E-26	-1.97	Cntnap5a	0.0003	7E-234	-2.72
Rassf9	2E-27	6E-24	-0.69	Gm29106	0.0411	6E-211	2.19
Slc38a5	3.8E-27	1E-23	-1.44	Vxn	0.0000	2E-207	-4.94
**Sult1a1**	7.6E-25	2E-21	1.18	Gm8251	0.0161	2E-198	2.61
Acot11	3E-23	6E-20	1.03	Niban1	0.0000	2E-198	2.72
Arap3	9.7E-23	2E-19	-1.07	Gm37960	0.0298	3E-196	-3.65

^*^Ctrl Control group, *logFC* log fold change

### Gene-set enrichment analysis

**Fig. 2:**
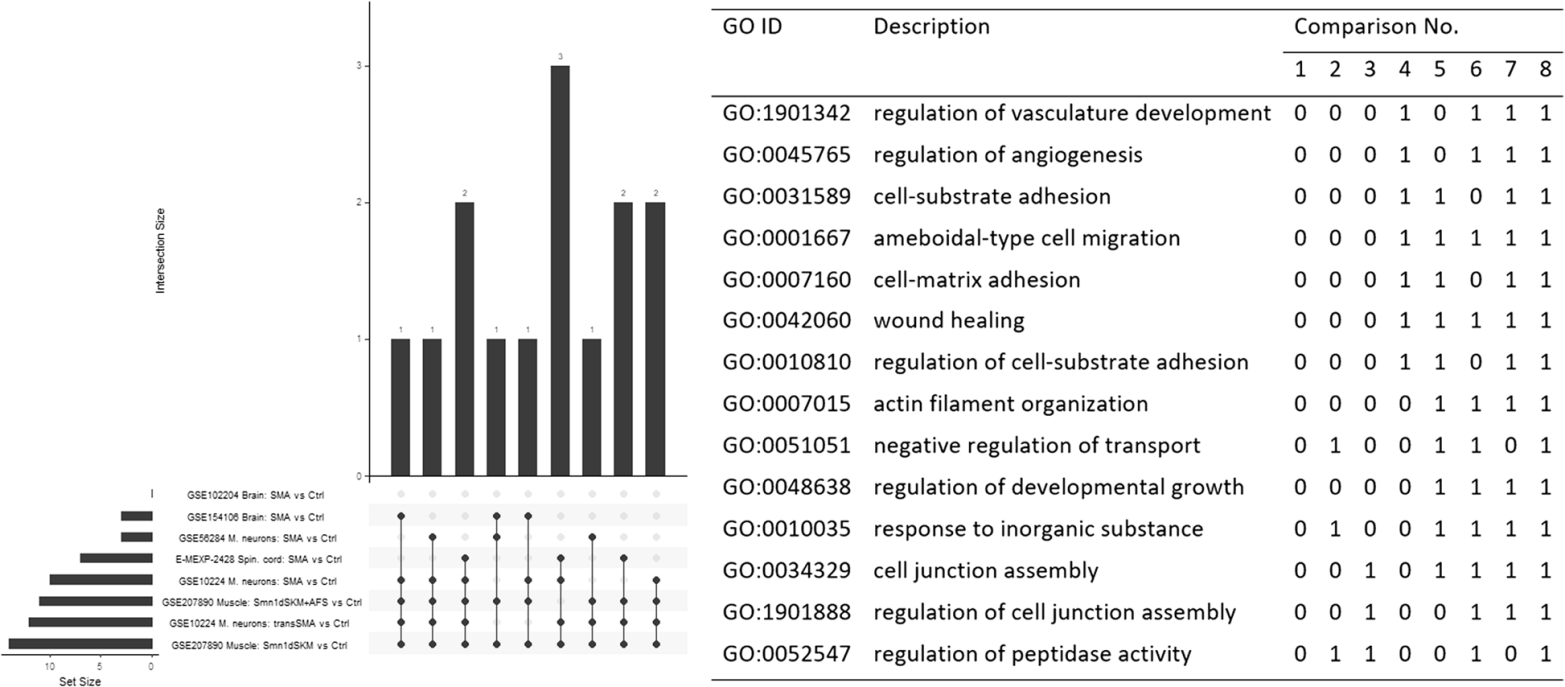
Upset plot (left) showing the number of GO terms related to biological processes (BP) that occur among the top 100 in at least four of the eight comparisons, and corresponding table (right) listing the 14 GO terms highlighted in the upset plot. The comparison number in the table corresponds to the order (from top to bottom) in the upset plot

For seven of the eight comparison significantly enriched GO terms were found after FDR-adjustment of enrichment *p*-values, the most ones in the domain of biological processes (Table 3). Only for data set GSE102204 (brain tissue), no significant terms were found. The bootstrap analysis showed a high robustness of the standard GO enrichment analysis, i.e. there were only moderate changes in the original ranking. There were larger overlaps between pairs of these lists, but only a smaller overlap between more than three lists. E.g., there were 14 GO terms related to BP that occurred in at least four of the eight comparisons (Fig. 2). Fourteen GO terms were also detected for MFs in at least four lists and 32 GO terms related to CCs (Fig SF2, Supplementary Data S1).

**Table 3:** Number of significantly enriched GO-terms from in the categories of biological processes, molecular function and cellular component in the eight comparisons

GEO or AE ID	Comparison	Tissue	# GO terms with pFDR < 0.05		
			Biological process	Molecular functions	Cellular component
GSE207890	Smn1δSKM + AFS versus Ctrl	Skeletal muscle	1,105	52	85
GSE207890	Smn1deltaSKM versus Ctrl	Skeletal muscle	1,056	48	77
GSE10224	SMA versus Ctrl	Motor neurons	1,709	186	141
GSE10224	transSMA versus Ctrl	Motor neurons	2,094	241	184
E-MEXP-2428	SMA versus Ctrl	Spinal cord	125	24	54
GSE154106	SMA versus Ctrl	Brain	574	87	74
GSE102204	SMA versus Ctrl	Brain	0	0	0
GSE56284	SMA versus Ctrl	Embryonic stem cell-derived motor neurons	958	111	130

Among the top 5 GO terms related to BPs for each comparison (ranked by the bootstrap approach, Table 4) were two which could directly be related to SMA, ‘muscle tissue development’ (GO:0060537) and ‘muscle cell development’ (GO:0055001). While most GO-BP terms occurred only once among the top 5, three terms occurred twice: ‘extracellular matrix organization’ (GO:0030198), ‘extracellular structure organization’ (GO:0043062), ‘external encapsulating structure organization’ (GO:0045229).

**Table 4:** Top 5 GO terms related to biological processes (BP) from the eight comparisons, including the number of genes associated with each gene, and their ranks from the original and bootstrap based GSEA

Data set / tissue	GO ID	Description	# Associated genes	pFDR (non boot)	Boot rank (Original rank)
**GSE207890** **Smn1δSKM + AFS** **vs Ctrl** **Skeletal muscle**	GO:0001667	Ameboidal-type cell migration	463	4.4*10^–7^	2 (1)
	GO:0034329	Cell junction assembly	477	4.4*10^–7^	2 (2)
	GO:0045765	Regulation of angiogenesis	469	2.0*10^–6^	2 (4)
	GO:0007015	Actin filament organization	489	1.4*10^–6^	4 (5)
	GO:0060537	Muscle tissue development	300	1.9*10^–6^	5 (3)
**GSE207890 Smn1δSKM vs Ctrl** **Skeletal muscle**	GO:0046631	alpha–beta T cell activation	216	2.6*10^–6^	1 (1)
	GO:0032943	Mononuclear cell proliferation	463	2.9*10^–6^	2 (4)
	GO:0052548	Regulation of endopeptidase activity	324	2.8*10^–6^	3 (3)
	GO:0007159	Leukocyte cell–cell adhesion	300	2.8*10^–6^	4 (5)
	GO:0001667	ameboidal-type cell migration	370	2.8*10^–6^	5 (6)
**GSE10224** **SMA vs Ctrl** **Motor neurons**	GO:0055002	Striated muscle cell development	63	2.0*10^–8^	2 (1)
	GO:0030239	Myofibril assembly	62	2.0*10^–8^	2 (2)
	GO:0031032	Actomyosin structure organization	175	2.0*10^–8^	2 (3)
	GO:0010927	Cellular component assembly involved in morphogenesis	105	2.0*10^–8^	4 (4)
	GO:0055001	Muscle cell development	193	2.0*10^–8^	5 (5)
**GSE10224 transSMA vs Ctrl Motor neurons**	GO:0030198	**Extracellular matrix organization**	258	7.8*10^–9^	2 (2)
	GO:0043062	**Extracellular structure organization**	259	7.8*10^–9^	2 (1)
	GO:0045229	**External encapsulating structure organization**	259	7.8*10^–9^	2 (3)
	GO:0010810	Regulation of cell-substrate adhesion	199	7.8*10^–9^	4 (4)
	GO:0031589	cell-substrate adhesion	195	7.8*10^–9^	5 (7)
**E-MEXP-2428 Spinal cord**	GO:0030198	**Extracellular matrix organization**	272	1.4*10^–7^	2 (1)
	GO:0043062	**Extracellular structure organization**	273	1.4*10^–7^	2 (2)
	GO:0045229	**External encapsulating structure organization**	273	1.4*10^–7^	2 (3)
	GO:0007272	Ensheathment of neurons	148	1.4*10^–7^	4 (4)
	GO:0001525	Angiogenesis	473	1.4*10^–7^	5 (6)
**GSE154106** **Brain**	GO:0071230	Cellular response to amino acid stimulus	154	7.8*10^–8^	3 (1)
	GO:0043200	Response to amino acid	169	7.8*10^–8^	3 (2)
	GO:0071229	Cellular response to acid chemical	167	7.8*10^–8^	3 (3)
	GO:0001101	Response to acid chemical	188	7.8*10^–8^	3 (4)
	GO:0060291	Long-term synaptic potentiation	236	7.8*10^–8^	3 (5)
**GSE102204** **Brain**	GO:0000070	Mitotic sister chromatid segregation	165	0.42	1 (3)
	GO:0000819	Sister chromatid segregation	203	0.25	2 (2)
	GO:0007059	Chromosome segregation	359	0.19	3 (1)
	GO:0098813	Nuclear chromosome segregation	73	0.67	4 (5)
	GO:0051304	Chromosome separation	116	0.42	5 (7)
**GSE56284 Embryonic stem cell-derived motor neurons**	GO:1,903,487	Regulation of lactation	21	1.5*10^–8^	2.5 (1)
	GO:1,903,489	Positive regulation of lactation	20	1.5*10^–8^	2.5 (3)
	GO:0007416	Synapse assembly	228	1.5*10^–8^	2.5 (2)
	GO:0007595	Lactation	81	1.5*10^–8^	2.5 (6)
	GO:0030534	Adult behavior	52	1.5*10^–8^	5.0 (4)

^*^Ctrl Control group, *GO* Gene Ontology

The top5 GO terms related to MFs and CCs are given in Supplementary Data S3 and S4. Overall, there was a bit more overlap compared to BP terms (Figure SF2, Supplementary Data S1). Specifically, these were the MF-terms ‘actin binding’ (GO:0003779), ‘actin filament binding’ (GO:0051015), ‘calmodulin binding’ (GO:0005516), ‘calcium ion binding’ (GO:0005509), ‘DNA-binding transcription activator activity, RNA polymerase II-specific’ (GO:0001228), ‘DNA-binding transcription activator activity’ (GO:0001216), ‘extracellular matrix structural constituent’ (GO:0005201), ‘structural molecule activity’ (GO:0005198), ‘signalling receptor regulator activity’ (GO:0030545), and the CC-terms membrane raft (GO:0045121), cell leading edge (GO:0031252), membrane microdomain (GO:0098857), collagen-containing extracellular matrix (GO:0062023), mitochondrial protein-containing complex (GO:0098798) which occurred more than once among the top 5.

From the analysis of KEGG and Reactome pathways, a moderate number of significantly enriched pathways emerged for most of the eight comparisons (Table ST3 Supplementary Data S1), except for the comparison in the brain tissue samples of data set GSE102204 which resulted in zero enriched pathways after FDR-adjustment. The largest number of enriched pathways was detected in the group comparisons of the motor neuron data set (GSE10224), ranging between 133 and 214 enriched pathways, while for the other group comparison the numbers ranged between 1ß and 87 enriched pathways.

Among the top5 from all comparisons, there were six KEGG pathways that were detected twice (Supplementary Data S5): ‘MicroRNAs in cancer’ (mmu05206), ‘Rap1 signaling pathway’ (mmu04015), ‘PI3K-Akt signaling pathway’ (mmu04151), ‘Focal adhesion’ (mmu04510) and ‘Neuroactive ligand-receptor interaction’ (mmu04080). From the enrichment analysis of Reactome pathways, seven pathways occurred more than once among the top5 of all comparisons, two of them even four time (Supplementary Data S6): ‘Hemostasis’ (R-MMU-109582) and ‘Signaling by Receptor Tyrosine Kinases’ (R-MMU-9006934). These other five pathways occurred twice each: ‘Developmental Biology’ (R-MMU-1266738), ‘Extracellular matrix organization’ (R-MMU-1474244), ‘Integrin cell surface interactions’ (R-MMU-216083), ‘mRNA Splicing’ (R-MMU-72163), and ‘Processing of Capped Intron-Containing Pre-mRNA’ (R-MMU-72203).

### Network reconstruction

**Fig. 3:**
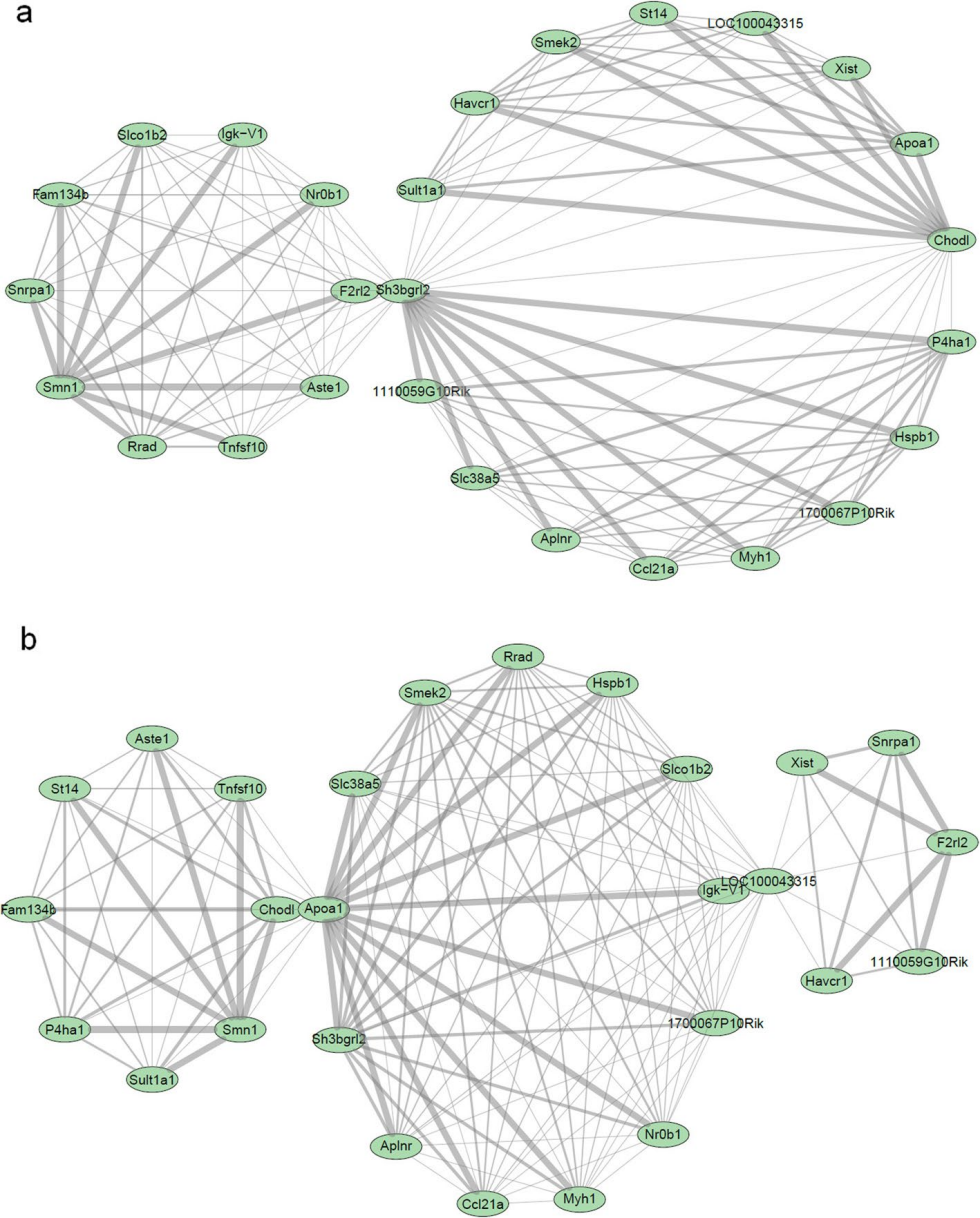
Networks constructed using the expression profiles from the GSE10224 data, SMA versus control, for the top 5 DEGs from all the datasets that were merged creating a gene set for each study group, where the top plot corresponds to SMA group and the plot at the bottom corresponds to control group. Nodes represents the genes and edges the interaction between them. The size and colour of the node shows the interaction size of the genes. The thickness of edge represents the correlation between the genes

The construction of networks was based on differential expression analyses from the eight different comparisons where the top5 genes were selected and merged to create a gene set for each comparison. We focus exemplarily on the networks arising from the data of data set GSE10224 with the comparison of SMA versus control and on connections with the Smn1 genes, the central player of SMA. For the SMA group (Fig. 3a), Smn1 has stronger correlation with genes Snrap1 (Small Nuclear Ribonucleoprotein Polypeptide A), Fam134b, Slco1b2, Igk-V1, Nr0b1, F2rl2, Aste1, Tnfsf10 and Rad, whereas in the control group Smn1 has strong correlation with genes Sult1a1, P4ha1, Fam134b, St14, Aste1, Tnfsf10 and Chodl (Fig. 3b). Furthermore, consistent patterns were observed between genes that includes Fam134b, Aste1 and Tnfsf10 across both SMA and control group. This could be because these genes have stable interactions or shared functional relationships. A strong change of correlations of the Smn1 gene with other genes between the disease group and the control group was also seen in the other data sets (Fig. SF3-SF9, Supplementary Data S1).

### Co-expression network analysis

**Fig. 4:**
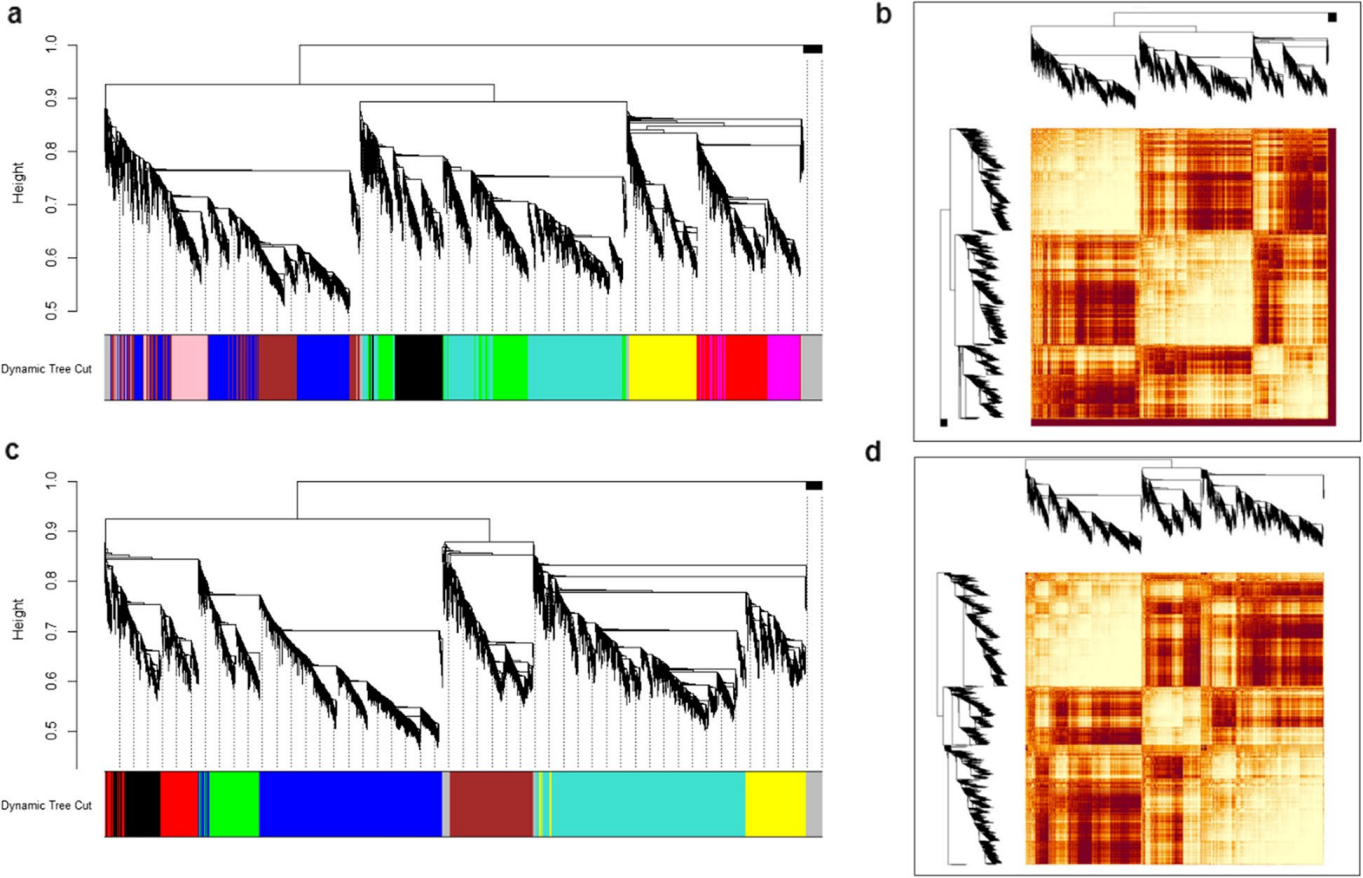
Co-expression modules and Topological Overlap Matrix (TOM) Plot associated with SMA (**a**, **b**) and Control (**c**, **d**) group of data set GSE102204. Plot **a** & **c**) WGCNA dendogram where the coloured bars towards the x-axis represent the module colours and y-axis represents the similarity between genes based on the height of the branches in the dendogram. Higher the value, greater the similarity between genes. SMA group has 9 modules and Control group has 7 modules excluding the grey module which correspond to the less correlated gene group. Plot **b** & **d**) TOM plot showing the topological overlap of the genes from data set GSE102204. Rows and columns represent the genes, darker the red colour higher the correlation between the genes. Their corresponding gene dendogram are towards the top and along the left of the plot

**Fig. 5:**
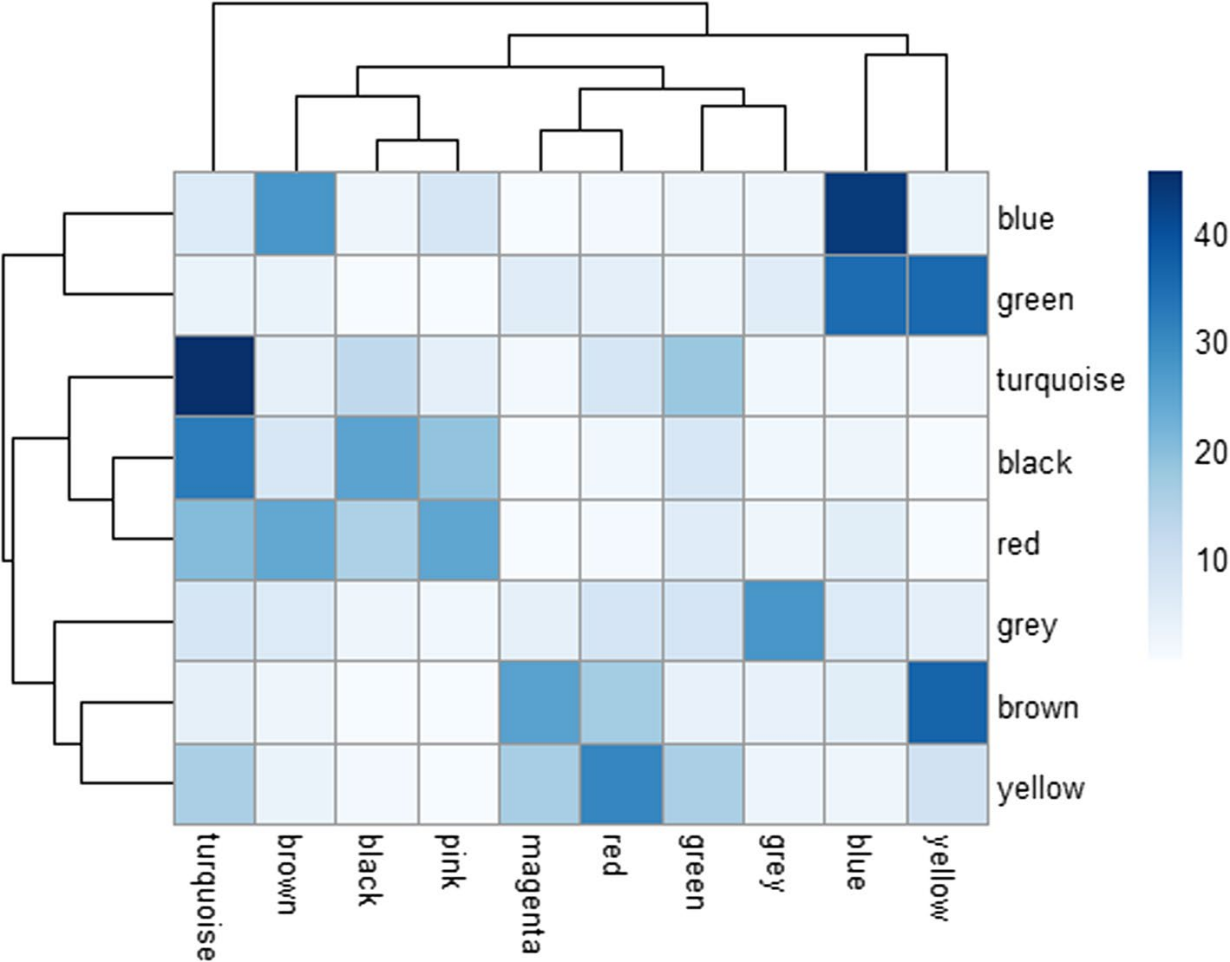
Heat map representing the amount of common genes in percentage between modules in the SMA and control group of data set GSE102204. Rows denote the control group and columns denote the SMA group

To understand more about the expression of genes across samples in each group for each study Weighted Gene Co-expression Network Analysis (WGCNA) analysis was performed. As a result, we identified co-expression modules for SMA and control groups where these modules represent clusters of genes with similar expression patterns across samples within each group (Fig. 4). In data set GSE102204 (brain tissue), SMA group, with17,097 genes, we identified nine modules excluding the grey module which corresponds to the less correlated group of genes. The size of each module varied between 1,020 (magenta module) and 2,800 (blue module) (Fig. 4a). Furthermore, in data set GSE102204, control group with 16,900 genes, a total of seven modules were identified with each module having genes between 951 (turquoise module) and 4,825 (blue module respectively) (Fig. 4b). To further understand these modules, top hub genes from each module were identified that denote the highly connected gene in each module. The top hub genes in the the SMA group of data set GSE102204 are Rrp1, Ints9, Ptgds, Nnt, 2010111I01Rik, Snx2, Gm26869, Agpat6 and Irak1. In the control group on the other hand with seven modules include Ube2v2, Apba1, Gm1043, Ids, Tm9sf2, Fkbp4 and Tmem183a. These hub genes could potentially represent important regulators of these modules. Furthermore, common genes among the modules between the control and SMA group of each study were analysed to better understand their role in SMA. In data set GSE102204 (Fig. 5), blue and turquoise modules have higher percentage of common genes and therefore over representation analysis was performed for these genes. Interestingly, genes like Smn1 and Snrpa1 which had strong correlations between genes (Fig. 5) are present in the blue and turquoise module respectively of both SMA and control group.

## Discussion

SMA has been studied at the level of the transcriptome in several studies, each with different settings and different approaches for data analysis. The idea of our meta-analysis was to aggregate the information from six selected studies by reanalysing the publicly available gene expression profiles in a harmonized way. Besides standard steps of analysis, such as differential expression analysis and gene-set enrichment analysis, we included network reconstruction and co-expression analysis which were not performed in the original studies. Overall, we could identify only small similarities between the results from the individual studies, however some differences which can be related to the different study settings such as mouse strains, types of organs and time points. Lists of differentially expressed genes are also provided in Supplementary Data S7. Potentially SMA-relevant genes were selected on different level: by differential expression analysis, by intersection analysis, and by network analysis. From these results, we highlight and discuss in the following some tissue specific and some commonly identified genes.

### Tissue-specific genes and gene-sets

Among the top10 genes from the two group comparisons in the skeletal muscle study (GSE207890), Apoa1 was related to have an important function in the skeletal muscle for several diseases [11] and particular in the context of neuronal injury [50]. Furthermore, Mdb5 was mentioned I the context of neurodevelopmental abnormalities [25]. While there was no overlap between the top10 of both comparisons, some of the top10 were also in the overall intersect of both comparisons (according pFDR < 0.05 and abs(logFC) > 2): 1700057K13Rik, Rrad, Fam134b, H60b, Sap30, Egln2.

The top10 arising from the analysis of the embryonic stem cell-derived motor neurons include the ASIC4 which was also reported as an important player in muscle functions [21], and Cdh7 which was reported to have a central role in neural development [19, 28]. In the motor neurons from the spinal cord (GSE10224), only one significant gene was identified, 1700067P10Rik, which was not mentioned so far in the context of neuro-degenerative or muscle-related diseases. Despite the weak effects, we observed in these data, we want to highlight St14, which occurred among the top10, and which was already mentioned in the context of muscular dystrophy in the 1980s [22, 26]. Furthermore, Sult1a1 was observed among the top10, which was reported in several studies on neurodegenerative diseases [8] and it was found among the top10 in one of the brain studies (GSE102204) as well.

Regarding the top10 from the group comparisons in brain samples (studies GSE102204 & GSE154106), we want to highlight the chemokine Ccl21a which was also related to neurological diseases [9] and Slc38a5 which has been shown to cause developmental delay in motor dysfunction [44]. Furthermore, Abca7 which was related to multiple neurological diseases, in particular cortical and hippocampal atrophy [45].

Among the GO-BP-terms that occurred only once among the top5 lists were several which can directly be linked to SMA: ‘Muscle tissue development’ (GO:0060537) in one of the skeletal muscle tissues, and ‘Striated muscle cell development’ (GO:0055002) and ‘Muscle cell development’ (GO:0055001) in motor neurons. Furthermore, there were two terms which can be related to neurological diseases: ‘Ensheathmeant of neurons’ (GO:0007272) in the spinal cord data and ‘Synapse assembly’ (GO:0007416) in the motor neurons derived from embryonic stem cells. While it is difficult to name a tissue-specific GO-MF-term which can be related to SMA or neurological diseases, there were several GO-CC-terms: ‘Striated muscle thin filament’ (GO0005865) in the motor neuron data and several terms related to synapses in the motor neurons derived from embryonic stem cells. From the top5 KEGG pathways, only ‘Axon guidance’ (mmu04360) in one of the brain tissues can be directly related to neurological diseases, and from the top5 Reactome pathways ‘Muscle contradiction’ (R-MMU-397014) and ‘Neuronal System’ (R-MMU-112316) make a direct sense in the context of SMA or neurological diseases.

### Tissue unspecific genes and gene-sets

From the 31 genes that showed some overlap between the eight comparisons, we want to highlight Mt2 (Metallothionein–1), a members of the Metallothionein family which is a metal binding protein involved in ion homeostasis, protection against oxidative stress and detoxification [15], and was is upregulated in motor neurons and skeletal muscle. Furthermore, Metallothioneins, Mt2 but also Mt1, have been linked to neuroprotection and neuro-regeneration which also promote activities such as neuronal survival and synaptic plasticity which in turn is important in the maintenance of neuronal function and integrity in neurodegenerative diseases [55] including SMA.

The three GO-BP terms that occurred among the top5 in motor neurons and spinal cord tissue, ‘Extracellular matrix organization’ (GO:00300198) has also been mentioned in the context of Duchenne muscular atrophy [59]. The GO-MF term ‘Actin binding’ (GO:0003779) which occurred three times among the top5 has been shown to play a role in sarcopenia, another muscular disease [20]. From the GO-CC enrichment analysis, ‘Collagen-containing extracellular matrix’ (GO:0062023) was among the top5 in motor neurons and skeletal muscle and was recently also mentioned in the context of intervertebral disc generation [61]. Furthermore the CC-term ‘mitochondrial protein-containing complex’ (GO:0098798) was mentioned to play a role in human juvenile Huntington’s disease, another neurological disease (Podvon et al., 2023).

Among the KEGG pathways that were among the top5 of the spinal cord and motor neuron analysis, was ‘Neuroactive ligand-receptor interaction’ (mmu04080) which was reported in the context of seizures and epilepsis [27,; Xiao et al., 2020). Interestingly, also two pathways which are related to cancer were among the top5: ‘MicroRNAs in cancer’ (mmu05206) and ‘PI3K-Akt signaling pathway’ (mmu04151). From the Reactome pathways that were among the top5 in several of the eight comparisons, ‘Hemostasis’ (R-MMU-109582) is naturally linked to muscle cells, and ‘Extracellular matrix organization’ (R-MMU-1474244) has regularly mentioned in the context of neurological disorder 51, 5].

### Comparison to original studies, reproducibility issues and limitations

A comparison of the top10 selected genes from our analysis with the findings reported in the original studies showed a very weak overlap. In fact, there were only four genes among our top10 lists that where directly names in the original publications of the six studies: Hspb1, Cdkn1a, Smn1 and Rgs5. Although many of the other genes selected by our analysis can be found in supplementary tables of the original publications, our study shows the weak reproducibility of original findings in transcriptomics studies if different reporting criteria are applied.

While reporting guidelines for microarray and RNAseq experiments where published long time ago – the MIAMI and the MINSEQE guidelines (https://www.ncbi.nlm.nih.gov/geo/info/MIAME.html) – the comparison of our meta-analysis with the original publications shows that an extension of these guidelines would be important for comparative meta-analyses. Specifically, the existing guidelines ask analysts to report methods for data processing and filtering of genes, but they do not make a recommendation which of the results should presented and in which form. For example, not all of the original publications provided lists of differentially expressed genes with *p*-values and fold changes but we did.

Furthermore, as an extension of the original analyses, we could identify interesting correlations between the top differentially expressed genes and modules of co-expressed genes by means of network reconstruction and co-expression analysis.

Although, a comparative meta-analysis, as presented here, has several advantages over individual studies, such as the harmonized way of analysis and the possibility of comparisons between study settings, there remain the same limitations as in single studies. Among these limitations are the risk for false negatives and false positives, and in general a risk for making arbitrary or biased selections. Therefore, we avoided to over-interpret the results and are aware that the studies included, here, have very small sample sizes. This summary of six transcriptomic studies can therefore not be seen as a proof of facts, but may help to further support findings of future omics studies on SMA.

## Supplementary Information

Supplementary Material 1. Data S1. Supplementary_Figures.

Supplementary Material 2. Data S2. WGCNA_SelectionCriterion.

Supplementary Material 3. Data S3. Top5_GO_MF.

Supplementary Material 4. Data S4. Top5_GO_CC.

Supplementary Material 5. Data S5. Top5_KEGG.

Supplementary Material 6. Data S6. Top5_Reactome.

Supplementary Material 7. Data S7. DEG.

## Data Availability

The datasets analysed during the current study are available in the Gene Expression Omnibus (GEO) and ArrayExpress (AE) repository, https://www.ncbi.nlm.nih.gov/geo/query/acc.cgi?acc=GSE207890 https://www.ncbi.nlm.nih.gov/geo/query/acc.cgi?acc=GSE154106 https://www.ncbi.nlm.nih.gov/geo/query/acc.cgi?acc=GSE102204 https://www.ncbi.nlm.nih.gov/geo/query/acc.cgi?acc=GSE56284 https://www.ncbi.nlm.nih.gov/geo/query/acc.cgi?acc=GSe10224 https://www.ebi.ac.uk/biostudies/arrayexpress/studies/E-MEXP-2428?query=E-MEXP-2428

## Abbreviations

AE: Array Express
Aste1: Asteroid Homolog 1
BP: Biological Process
Cacna2d2: Calcium Voltage-Gated Channel Auxiliary Subunit Alpha2delta 2
CC: Cellular Component
cDNA: Complementary DNA
Chodl: Chondrolectin
DEGs: Differentially Expressed Genes
ES: Enrichment score
F2rl2: Coagulation Factor II Thrombin Receptor Like 2
Fam134b: Reticulophagy Regulator 1
GEO: Gene Expression Omnibus
GO: Gene Ontology
GSEA: Gene-Set Enrichment Analysis
Hspb1: Heat Shock Protein Family B Member 1
Igk-V1: Immunoglobulin Kappa Variable 1
KEGG: Kyoto Encyclopedia of genes and genomes
MF: Molecular Function
mRNA: Messenger RNA
Mt1: Metallothionein 1A
Mt2: Metallothionein 2A
Nr0b1: Nuclear Receptor Subfamily 0 Group B Member 1
P4ha1: Prolyl 4-Hydroxylase Subunit Alpha 1
PRISMA: Preferred Reporting Items for Systematic Reviews and Meta-Analyses
Rad: Ras Related Glycolysis Inhibitor And Calcium Channel Regulator
Slco1b2: Solute carrier organic anion transporter family, member 1b2
SMA: Spinal Muscular Atrophy
Snrpa1: Small Nuclear Ribonucleoprotein Polypeptide A
SRA: Sequence Read Archive
St14: Transmembrane Serine Protease Matriptase
Sult1a1: Sulfotransferase Family 1A Member 1
Tnfsf10: TNF Superfamily Member 10
TOM: Topological Overlap Matrix
WGCNA: Weighted Gene Co-expression Network Analysis
