# Supplementary material for: Comparative meta-analysis of transcriptomic studies in spinal muscular atrophy: comparison between tissues and mouse models
Source: BMC Med Genomics. 2024 Nov 12;17:266. doi: 10.1186/s12920-024-02040-0 (PMC11555813; doi:10.1186/s12920-024-02040-0)
Supplement: Supplementary file 1 — Supplementary Material 1. Data S1. Supplementary_Figures. [file 12920_2024_2040_MOESM1_ESM.pptx]

## Slide 1
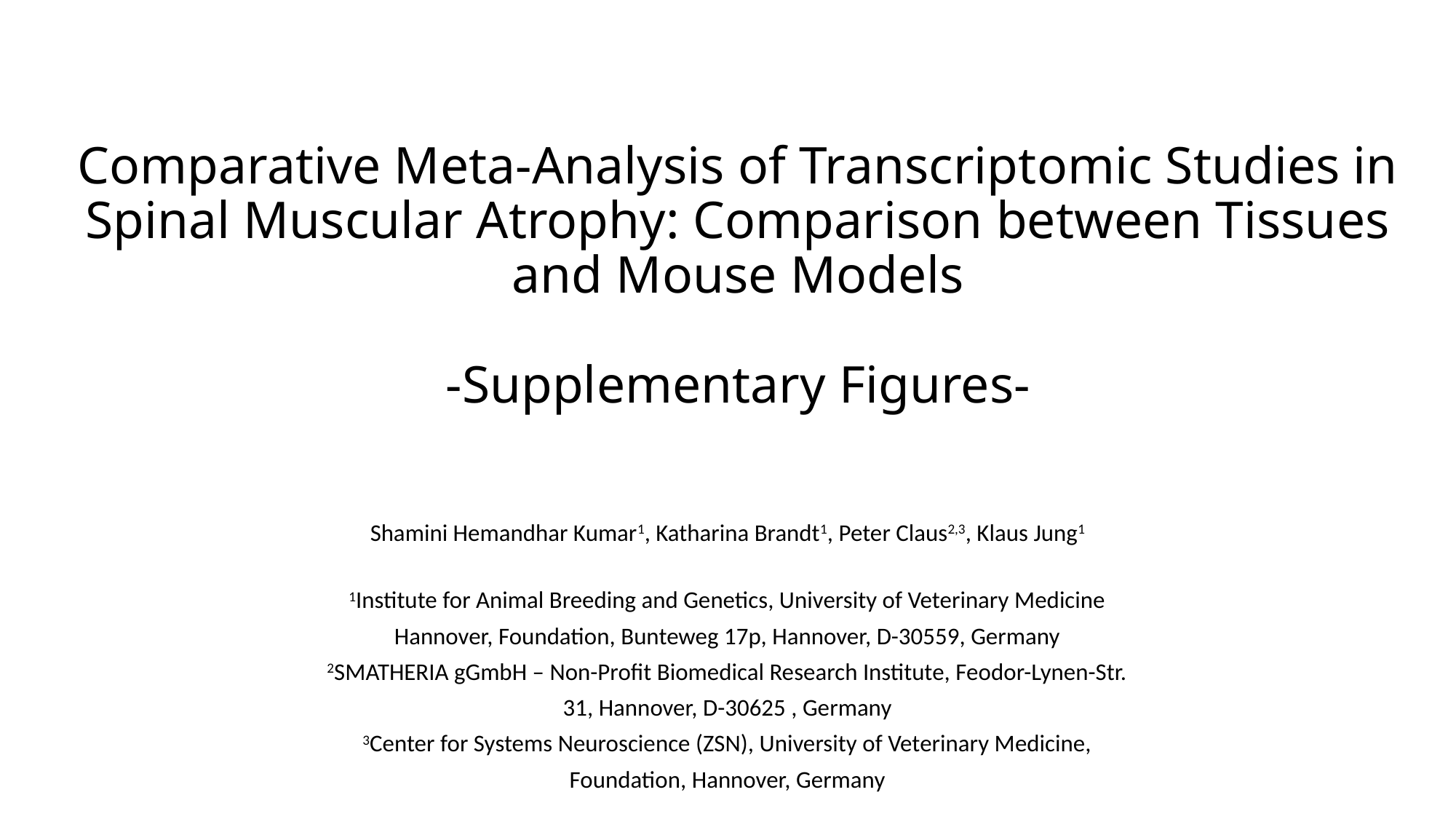

# Comparative Meta-Analysis of Transcriptomic Studies in Spinal Muscular Atrophy: Comparison between Tissues and Mouse Models -Supplementary Figures-
Shamini Hemandhar Kumar1, Katharina Brandt1, Peter Claus2,3, Klaus Jung1
1Institute for Animal Breeding and Genetics, University of Veterinary Medicine
Hannover, Foundation, Bunteweg 17p, Hannover, D-30559, Germany
2SMATHERIA gGmbH – Non-Profit Biomedical Research Institute, Feodor-Lynen-Str.
31, Hannover, D-30625 , Germany
3Center for Systems Neuroscience (ZSN), University of Veterinary Medicine,
Foundation, Hannover, Germany

## Slide 2
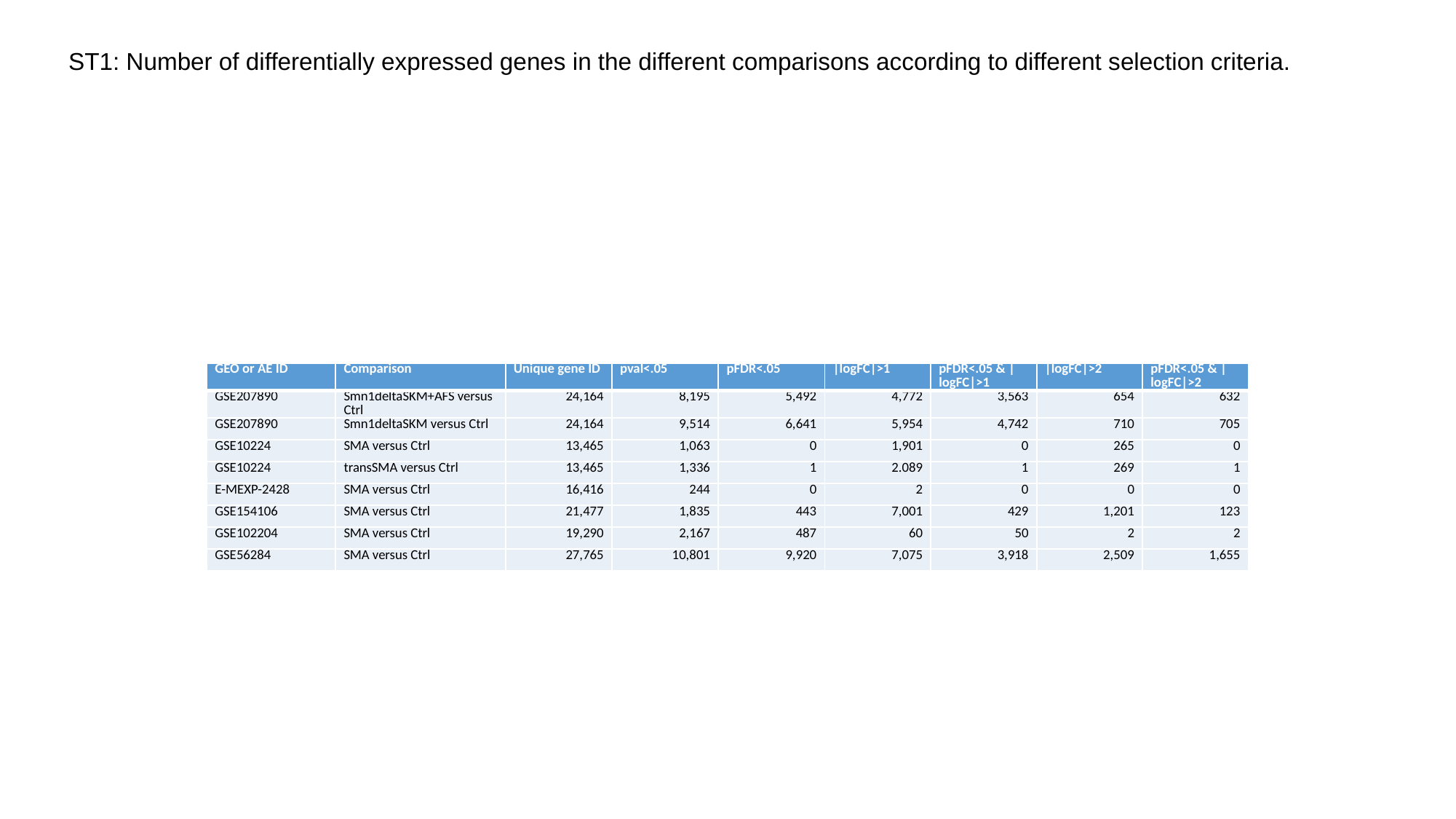

# ST1: Number of differentially expressed genes in the different comparisons according to different selection criteria.
| GEO or AE ID | Comparison | Unique gene ID | pval<.05 | pFDR<.05 | |logFC|>1 | pFDR<.05 & |logFC|>1 | |logFC|>2 | pFDR<.05 & |logFC|>2 |
| --- | --- | --- | --- | --- | --- | --- | --- | --- |
| GSE207890 | Smn1deltaSKM+AFS versus Ctrl | 24,164 | 8,195 | 5,492 | 4,772 | 3,563 | 654 | 632 |
| GSE207890 | Smn1deltaSKM versus Ctrl | 24,164 | 9,514 | 6,641 | 5,954 | 4,742 | 710 | 705 |
| GSE10224 | SMA versus Ctrl | 13,465 | 1,063 | 0 | 1,901 | 0 | 265 | 0 |
| GSE10224 | transSMA versus Ctrl | 13,465 | 1,336 | 1 | 2.089 | 1 | 269 | 1 |
| E-MEXP-2428 | SMA versus Ctrl | 16,416 | 244 | 0 | 2 | 0 | 0 | 0 |
| GSE154106 | SMA versus Ctrl | 21,477 | 1,835 | 443 | 7,001 | 429 | 1,201 | 123 |
| GSE102204 | SMA versus Ctrl | 19,290 | 2,167 | 487 | 60 | 50 | 2 | 2 |
| GSE56284 | SMA versus Ctrl | 27,765 | 10,801 | 9,920 | 7,075 | 3,918 | 2,509 | 1,655 |

## Slide 3
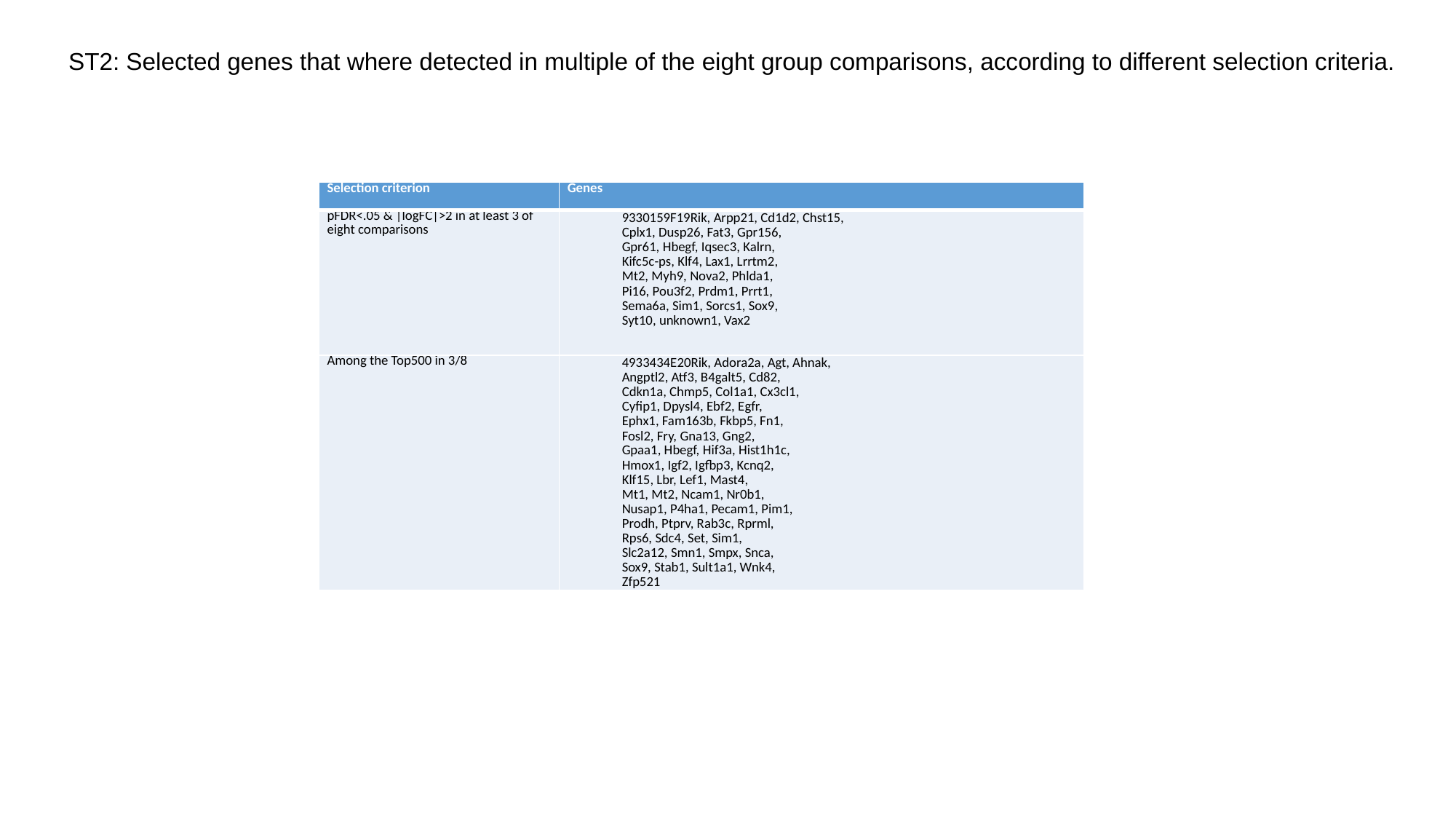

# ST2: Selected genes that where detected in multiple of the eight group comparisons, according to different selection criteria.
| Selection criterion | Genes |
| --- | --- |
| pFDR<.05 & |logFC|>2 in at least 3 of eight comparisons | 9330159F19Rik, Arpp21, Cd1d2, Chst15, Cplx1, Dusp26, Fat3, Gpr156, Gpr61, Hbegf, Iqsec3, Kalrn, Kifc5c-ps, Klf4, Lax1, Lrrtm2, Mt2, Myh9, Nova2, Phlda1, Pi16, Pou3f2, Prdm1, Prrt1, Sema6a, Sim1, Sorcs1, Sox9, Syt10, unknown1, Vax2 |
| Among the Top500 in 3/8 | 4933434E20Rik, Adora2a, Agt, Ahnak, Angptl2, Atf3, B4galt5, Cd82, Cdkn1a, Chmp5, Col1a1, Cx3cl1, Cyfip1, Dpysl4, Ebf2, Egfr, Ephx1, Fam163b, Fkbp5, Fn1, Fosl2, Fry, Gna13, Gng2, Gpaa1, Hbegf, Hif3a, Hist1h1c, Hmox1, Igf2, Igfbp3, Kcnq2, Klf15, Lbr, Lef1, Mast4, Mt1, Mt2, Ncam1, Nr0b1, Nusap1, P4ha1, Pecam1, Pim1, Prodh, Ptprv, Rab3c, Rprml, Rps6, Sdc4, Set, Sim1, Slc2a12, Smn1, Smpx, Snca, Sox9, Stab1, Sult1a1, Wnk4, Zfp521 |

## Slide 4
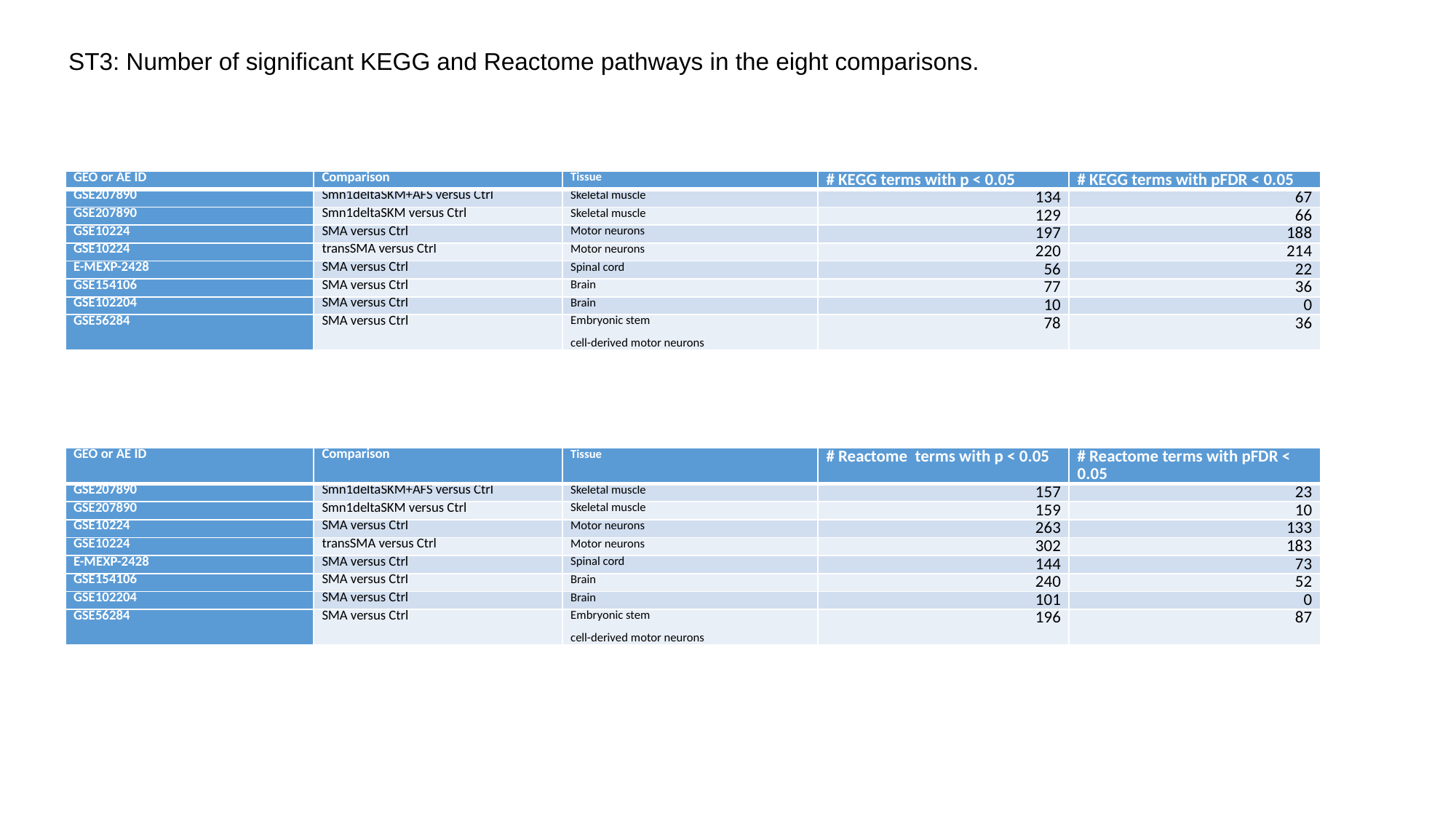

# ST3: Number of significant KEGG and Reactome pathways in the eight comparisons.
| GEO or AE ID | Comparison | Tissue | # KEGG terms with p < 0.05 | # KEGG terms with pFDR < 0.05 |
| --- | --- | --- | --- | --- |
| GSE207890 | Smn1deltaSKM+AFS versus Ctrl | Skeletal muscle | 134 | 67 |
| GSE207890 | Smn1deltaSKM versus Ctrl | Skeletal muscle | 129 | 66 |
| GSE10224 | SMA versus Ctrl | Motor neurons | 197 | 188 |
| GSE10224 | transSMA versus Ctrl | Motor neurons | 220 | 214 |
| E-MEXP-2428 | SMA versus Ctrl | Spinal cord | 56 | 22 |
| GSE154106 | SMA versus Ctrl | Brain | 77 | 36 |
| GSE102204 | SMA versus Ctrl | Brain | 10 | 0 |
| GSE56284 | SMA versus Ctrl | Embryonic stem cell-derived motor neurons | 78 | 36 |
| GEO or AE ID | Comparison | Tissue | # Reactome terms with p < 0.05 | # Reactome terms with pFDR < 0.05 |
| --- | --- | --- | --- | --- |
| GSE207890 | Smn1deltaSKM+AFS versus Ctrl | Skeletal muscle | 157 | 23 |
| GSE207890 | Smn1deltaSKM versus Ctrl | Skeletal muscle | 159 | 10 |
| GSE10224 | SMA versus Ctrl | Motor neurons | 263 | 133 |
| GSE10224 | transSMA versus Ctrl | Motor neurons | 302 | 183 |
| E-MEXP-2428 | SMA versus Ctrl | Spinal cord | 144 | 73 |
| GSE154106 | SMA versus Ctrl | Brain | 240 | 52 |
| GSE102204 | SMA versus Ctrl | Brain | 101 | 0 |
| GSE56284 | SMA versus Ctrl | Embryonic stem cell-derived motor neurons | 196 | 87 |

## Slide 5
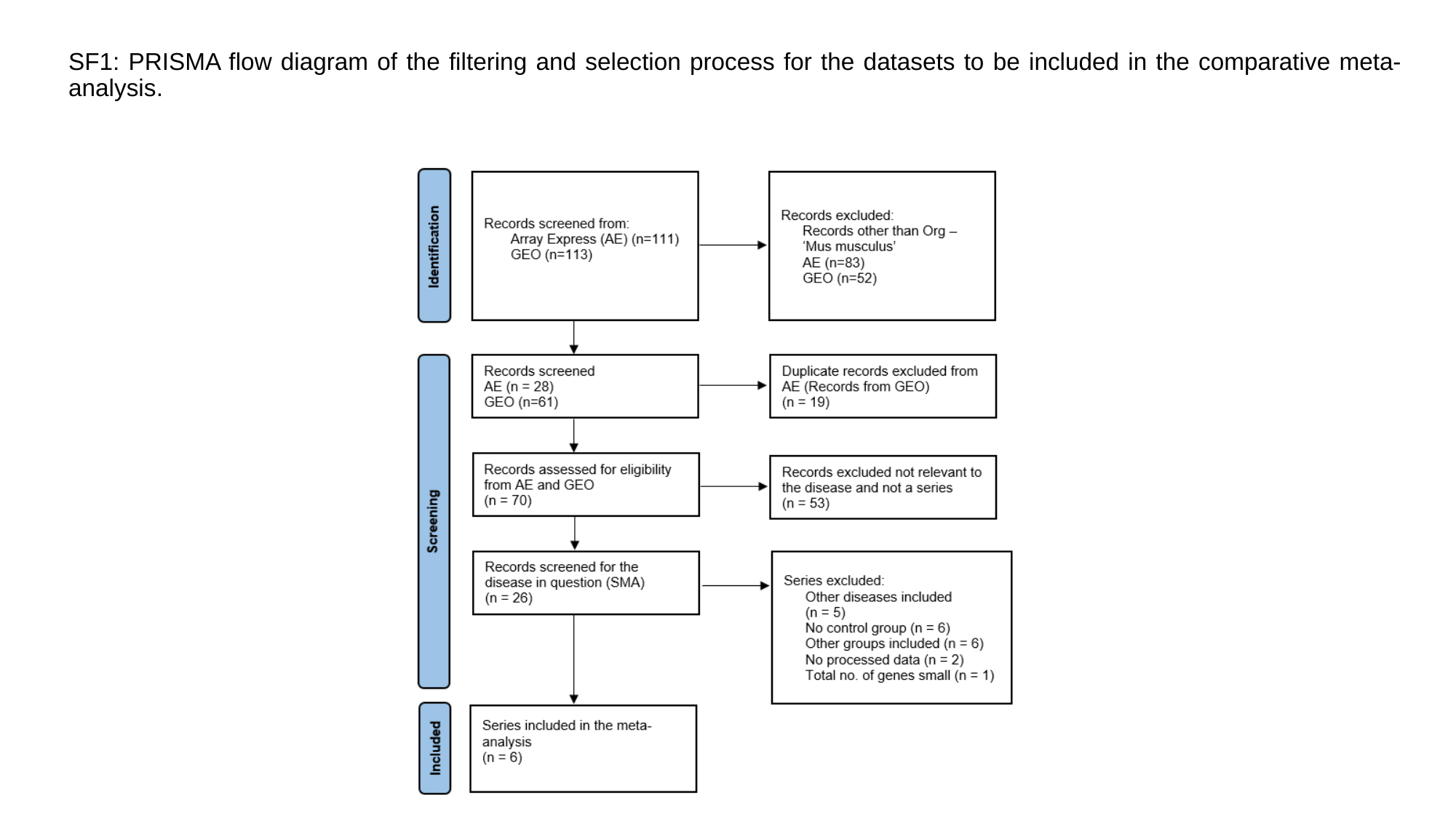

# SF1: PRISMA flow diagram of the filtering and selection process for the datasets to be included in the comparative meta- analysis.

## Slide 6
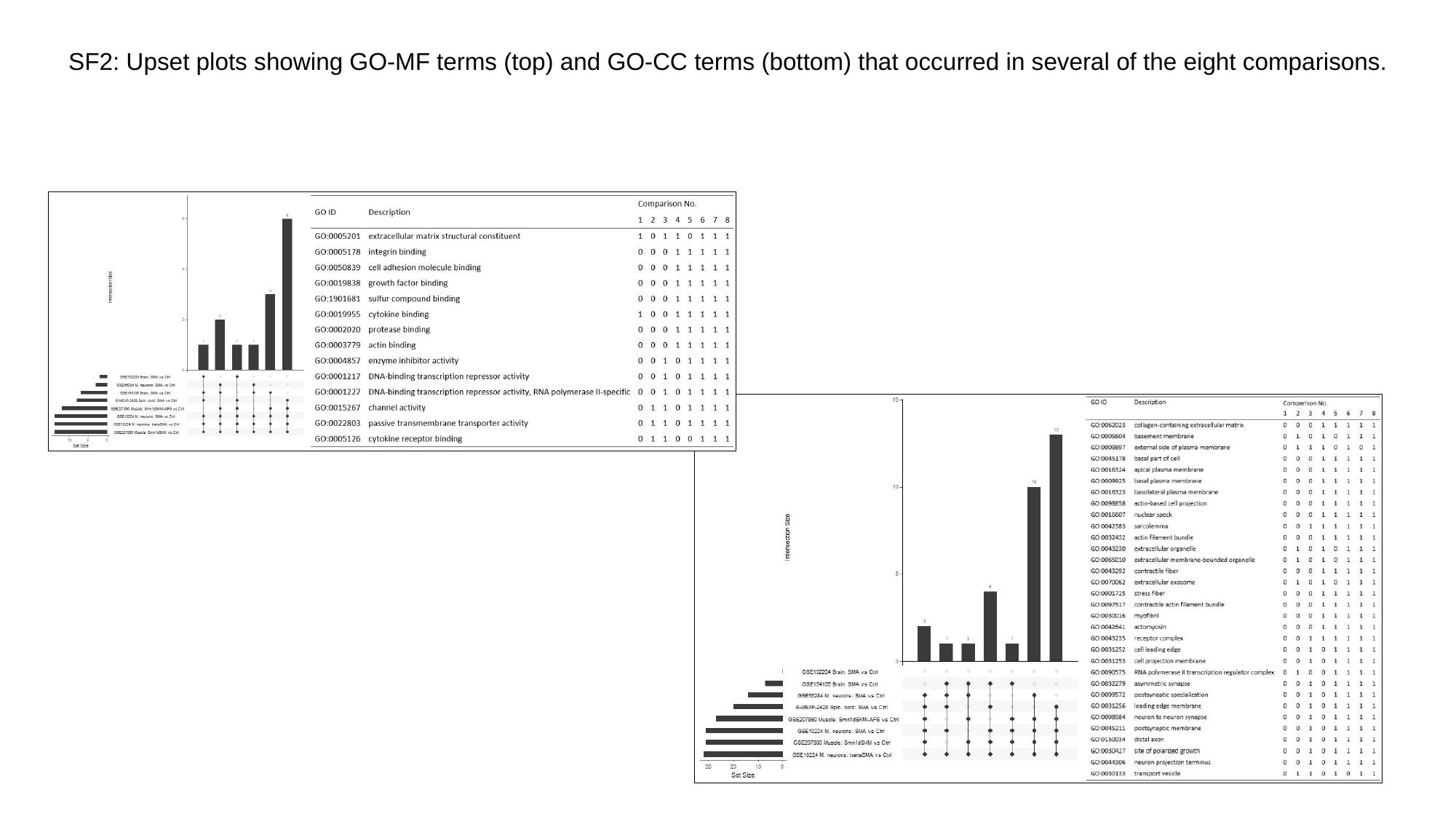

# SF2: Upset plots showing GO-MF terms (top) and GO-CC terms (bottom) that occurred in several of the eight comparisons.

## Slide 7
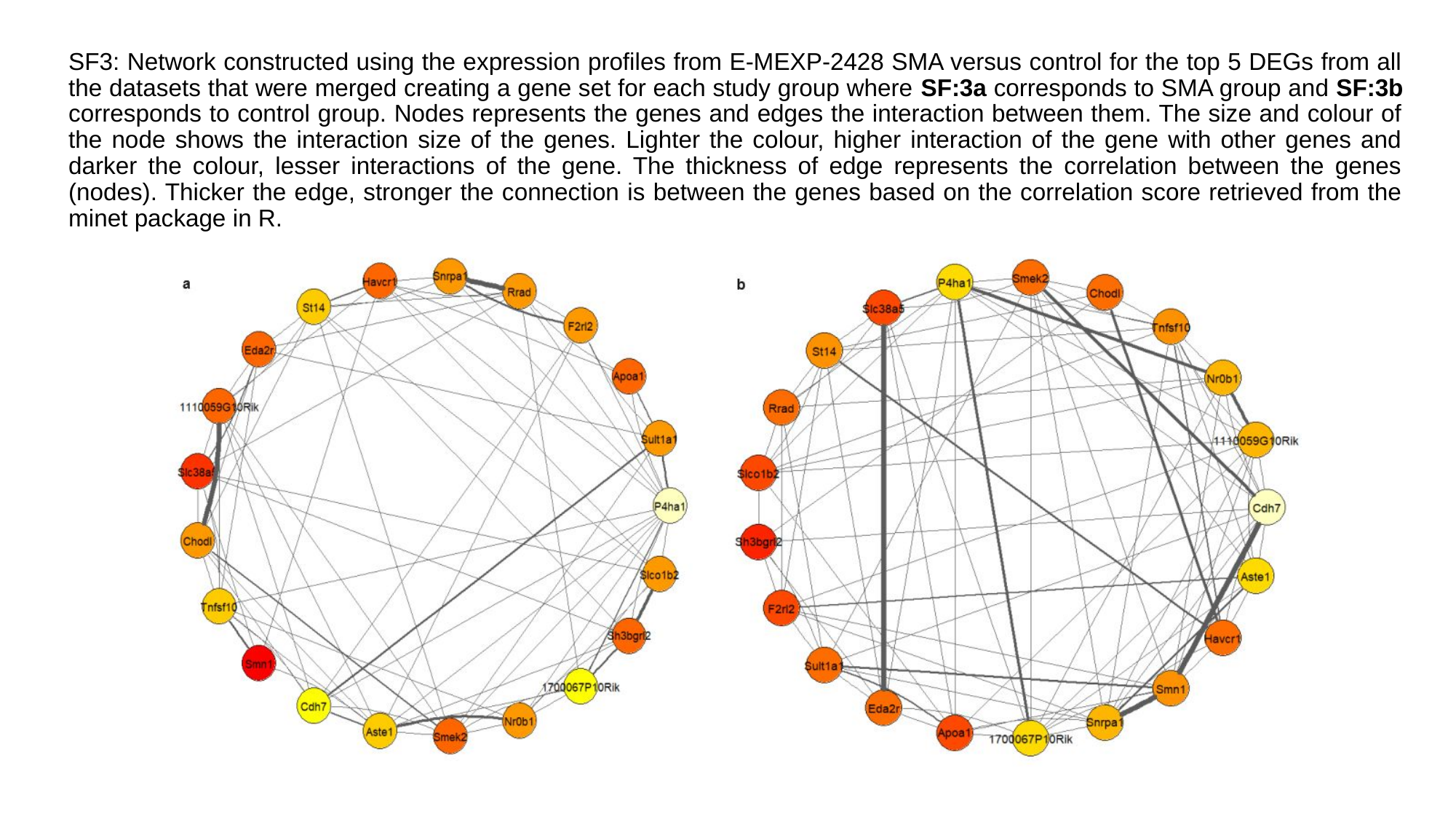

# SF3: Network constructed using the expression profiles from E-MEXP-2428 SMA versus control for the top 5 DEGs from all the datasets that were merged creating a gene set for each study group where SF:3a corresponds to SMA group and SF:3b corresponds to control group. Nodes represents the genes and edges the interaction between them. The size and colour of the node shows the interaction size of the genes. Lighter the colour, higher interaction of the gene with other genes and darker the colour, lesser interactions of the gene. The thickness of edge represents the correlation between the genes (nodes). Thicker the edge, stronger the connection is between the genes based on the correlation score retrieved from the minet package in R.

## Slide 8
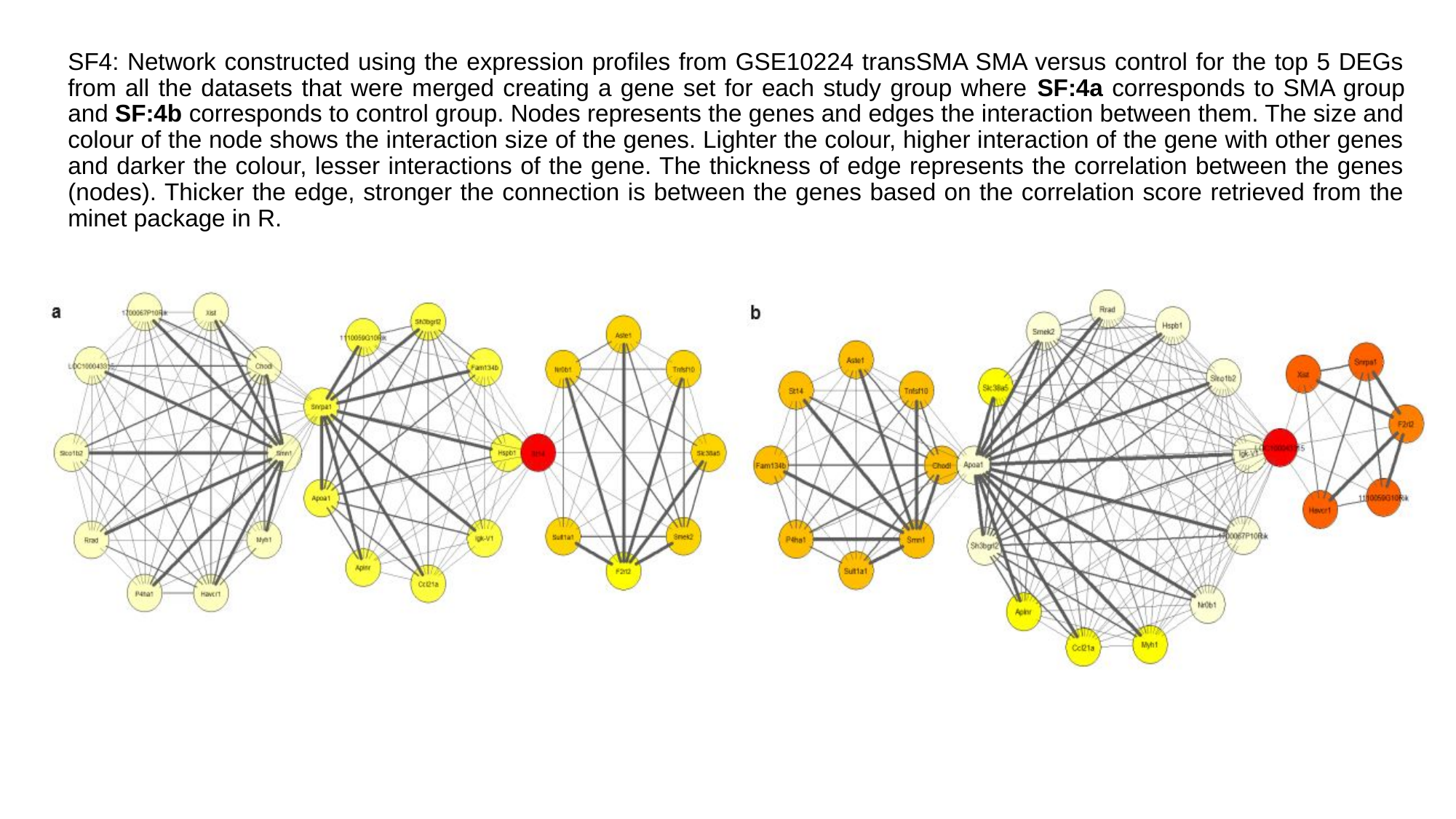

# SF4: Network constructed using the expression profiles from GSE10224 transSMA SMA versus control for the top 5 DEGs from all the datasets that were merged creating a gene set for each study group where SF:4a corresponds to SMA group and SF:4b corresponds to control group. Nodes represents the genes and edges the interaction between them. The size and colour of the node shows the interaction size of the genes. Lighter the colour, higher interaction of the gene with other genes and darker the colour, lesser interactions of the gene. The thickness of edge represents the correlation between the genes (nodes). Thicker the edge, stronger the connection is between the genes based on the correlation score retrieved from the minet package in R.

## Slide 9
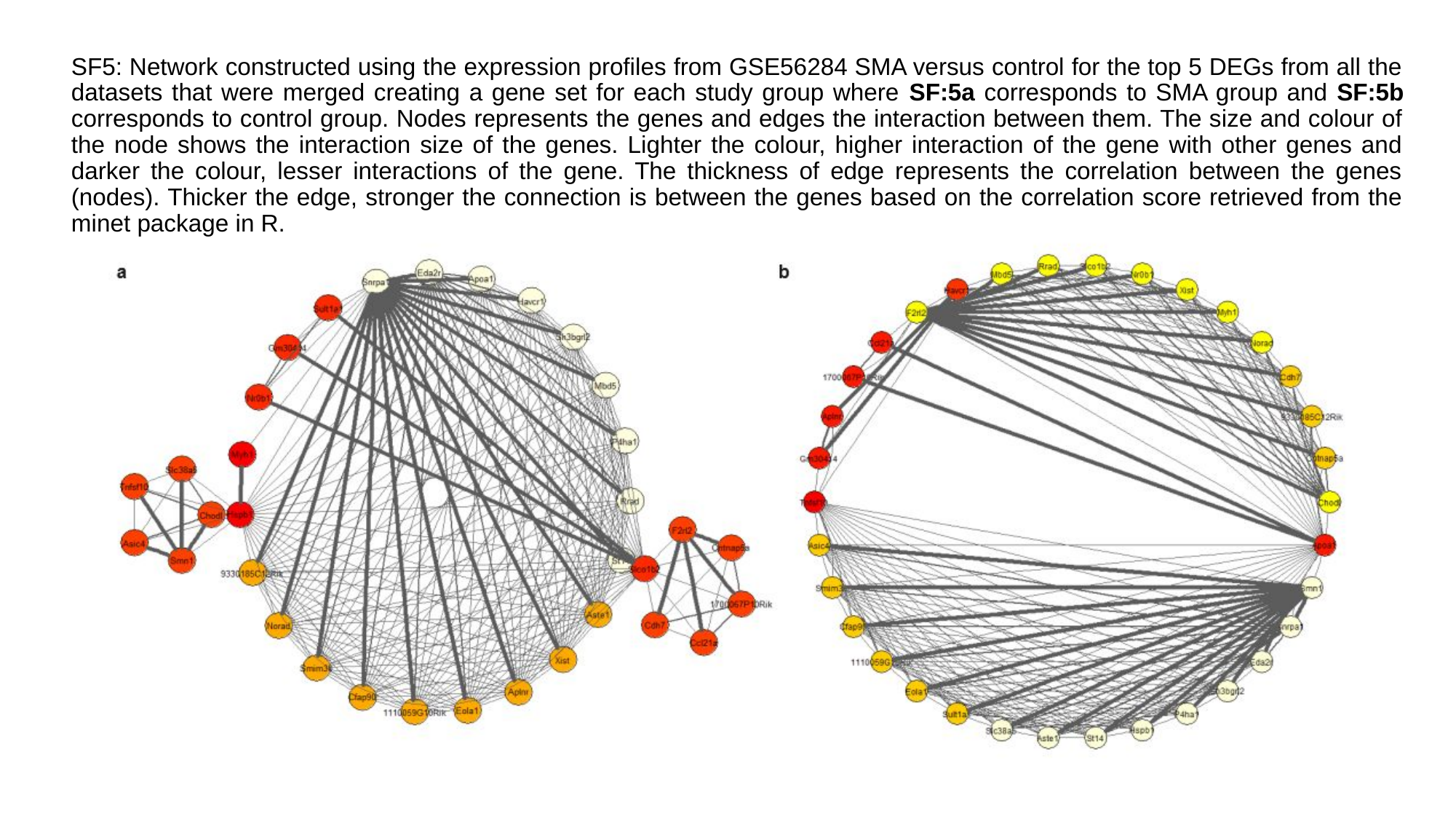

SF5: Network constructed using the expression profiles from GSE56284 SMA versus control for the top 5 DEGs from all the datasets that were merged creating a gene set for each study group where SF:5a corresponds to SMA group and SF:5b corresponds to control group. Nodes represents the genes and edges the interaction between them. The size and colour of the node shows the interaction size of the genes. Lighter the colour, higher interaction of the gene with other genes and darker the colour, lesser interactions of the gene. The thickness of edge represents the correlation between the genes (nodes). Thicker the edge, stronger the connection is between the genes based on the correlation score retrieved from the minet package in R.

## Slide 10
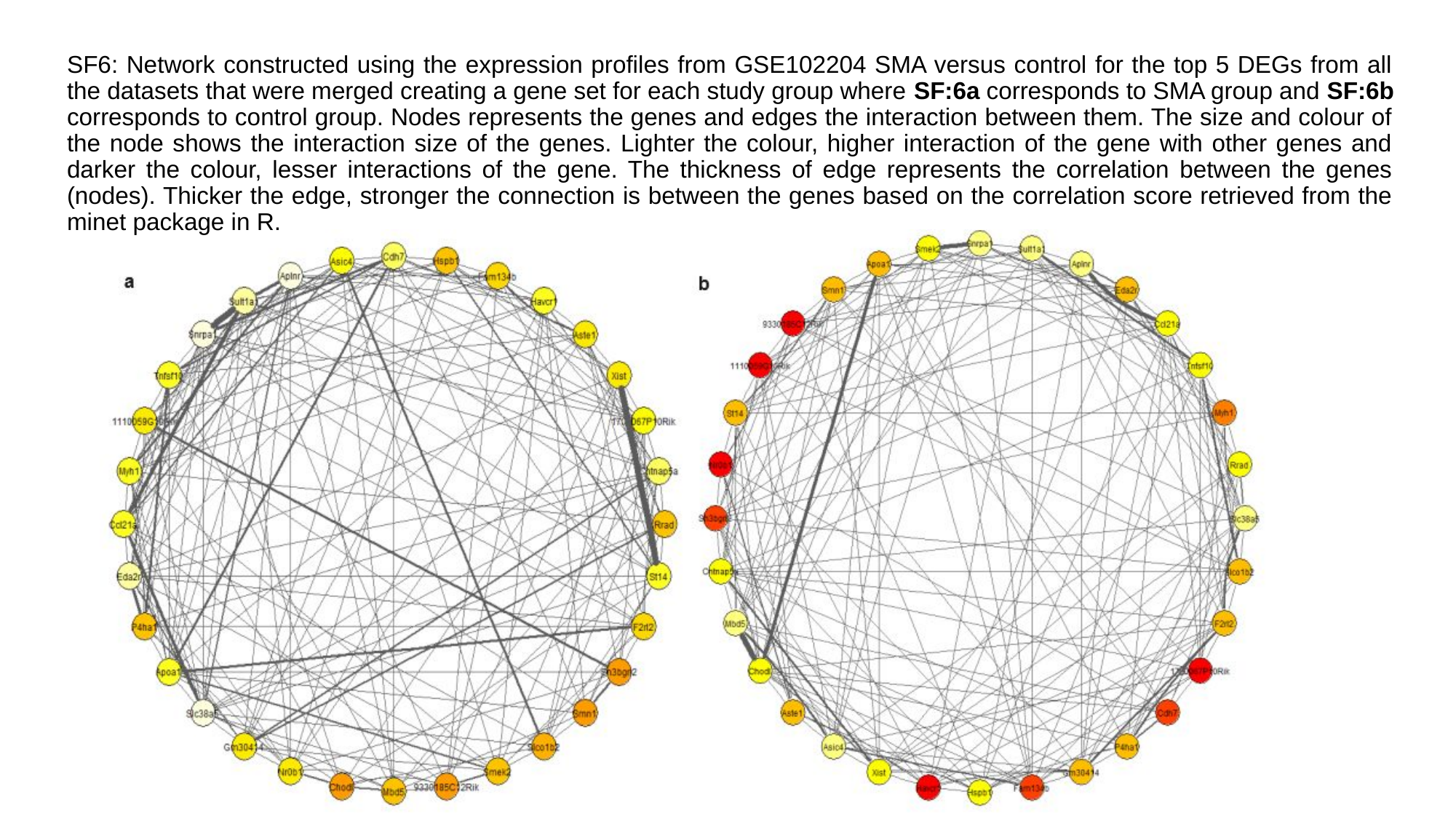

SF6: Network constructed using the expression profiles from GSE102204 SMA versus control for the top 5 DEGs from all the datasets that were merged creating a gene set for each study group where SF:6a corresponds to SMA group and SF:6b corresponds to control group. Nodes represents the genes and edges the interaction between them. The size and colour of the node shows the interaction size of the genes. Lighter the colour, higher interaction of the gene with other genes and darker the colour, lesser interactions of the gene. The thickness of edge represents the correlation between the genes (nodes). Thicker the edge, stronger the connection is between the genes based on the correlation score retrieved from the minet package in R.

## Slide 11
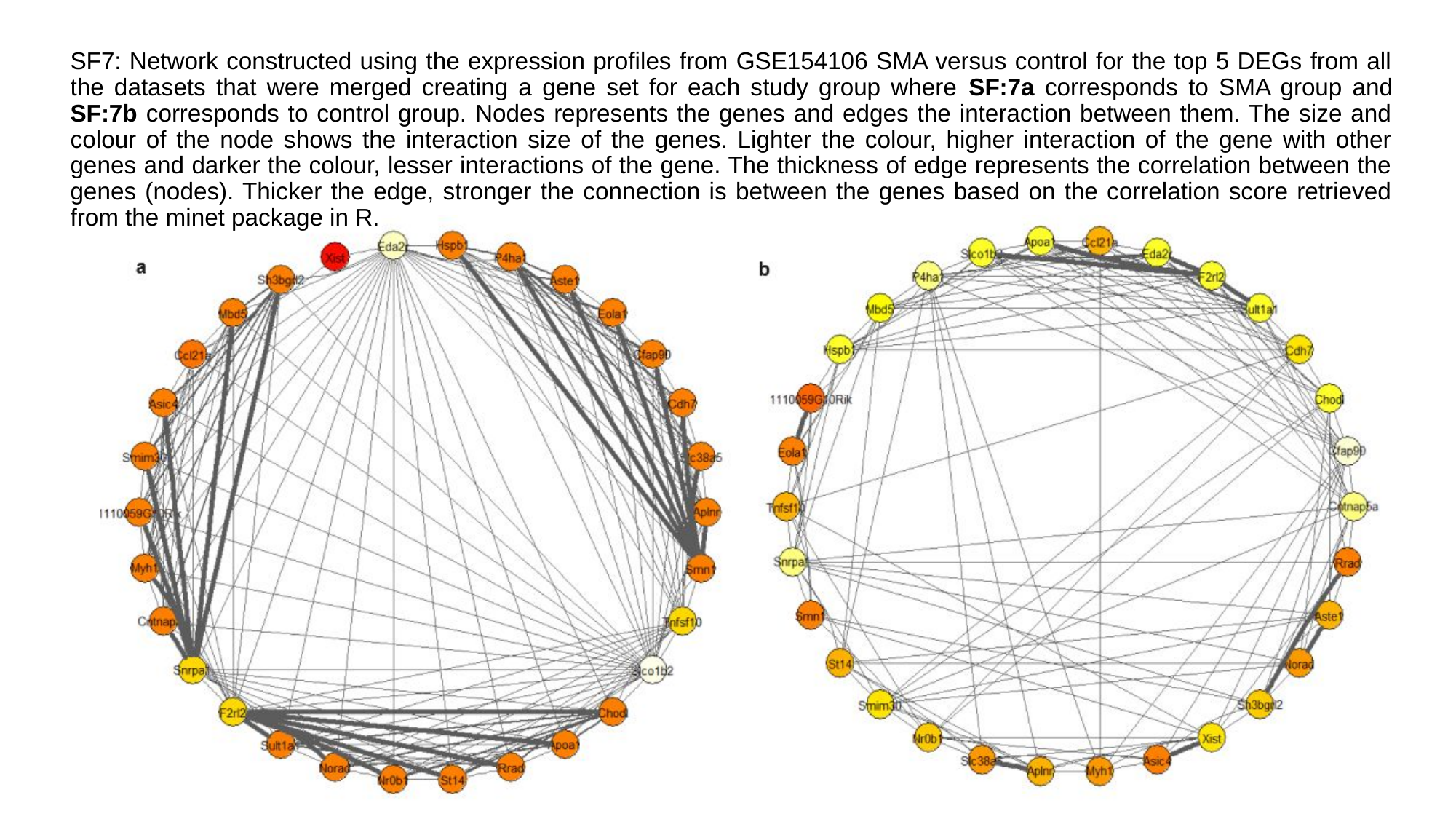

SF7: Network constructed using the expression profiles from GSE154106 SMA versus control for the top 5 DEGs from all the datasets that were merged creating a gene set for each study group where SF:7a corresponds to SMA group and SF:7b corresponds to control group. Nodes represents the genes and edges the interaction between them. The size and colour of the node shows the interaction size of the genes. Lighter the colour, higher interaction of the gene with other genes and darker the colour, lesser interactions of the gene. The thickness of edge represents the correlation between the genes (nodes). Thicker the edge, stronger the connection is between the genes based on the correlation score retrieved from the minet package in R.

## Slide 12
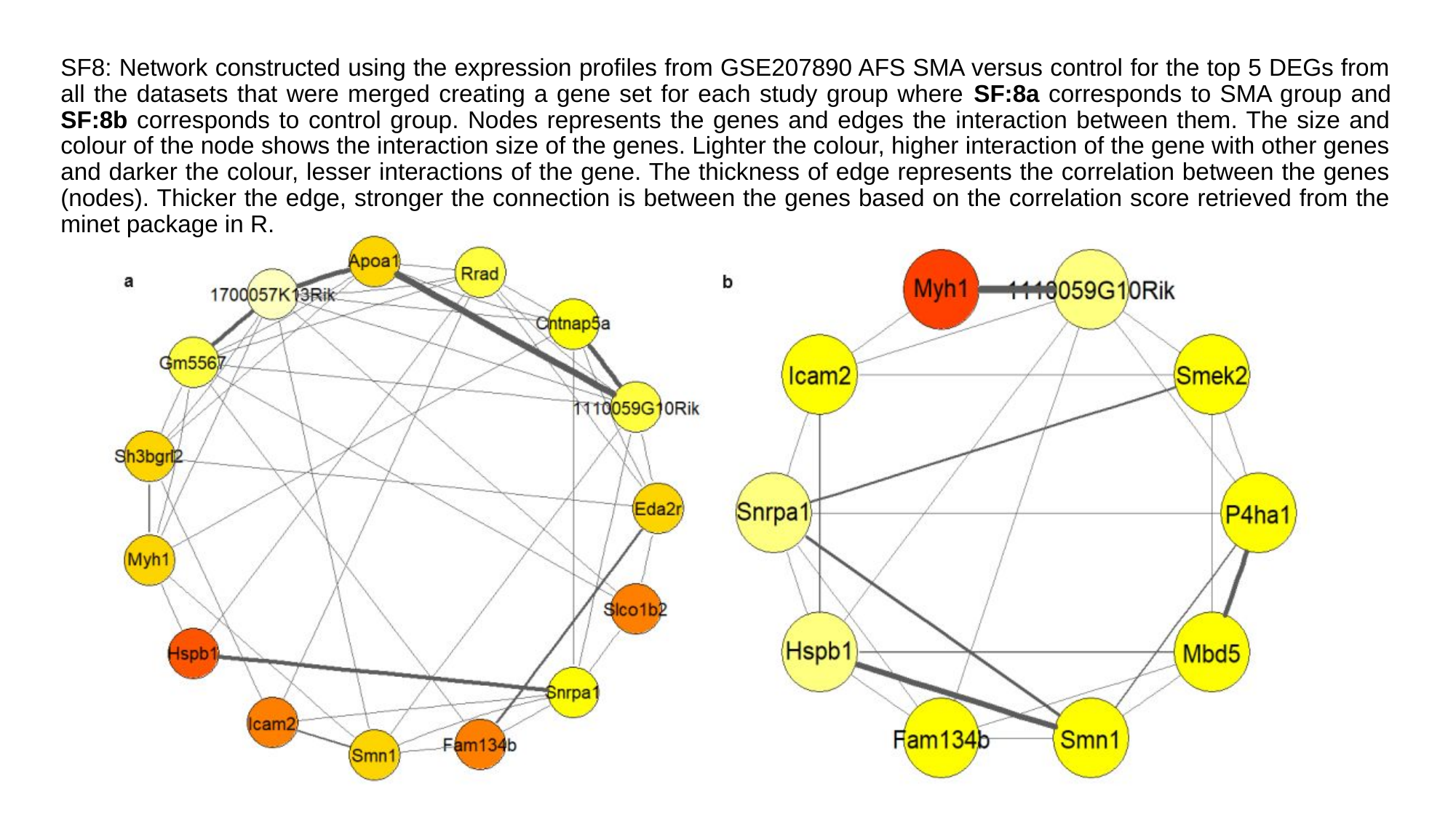

SF8: Network constructed using the expression profiles from GSE207890 AFS SMA versus control for the top 5 DEGs from all the datasets that were merged creating a gene set for each study group where SF:8a corresponds to SMA group and SF:8b corresponds to control group. Nodes represents the genes and edges the interaction between them. The size and colour of the node shows the interaction size of the genes. Lighter the colour, higher interaction of the gene with other genes and darker the colour, lesser interactions of the gene. The thickness of edge represents the correlation between the genes (nodes). Thicker the edge, stronger the connection is between the genes based on the correlation score retrieved from the minet package in R.

## Slide 13
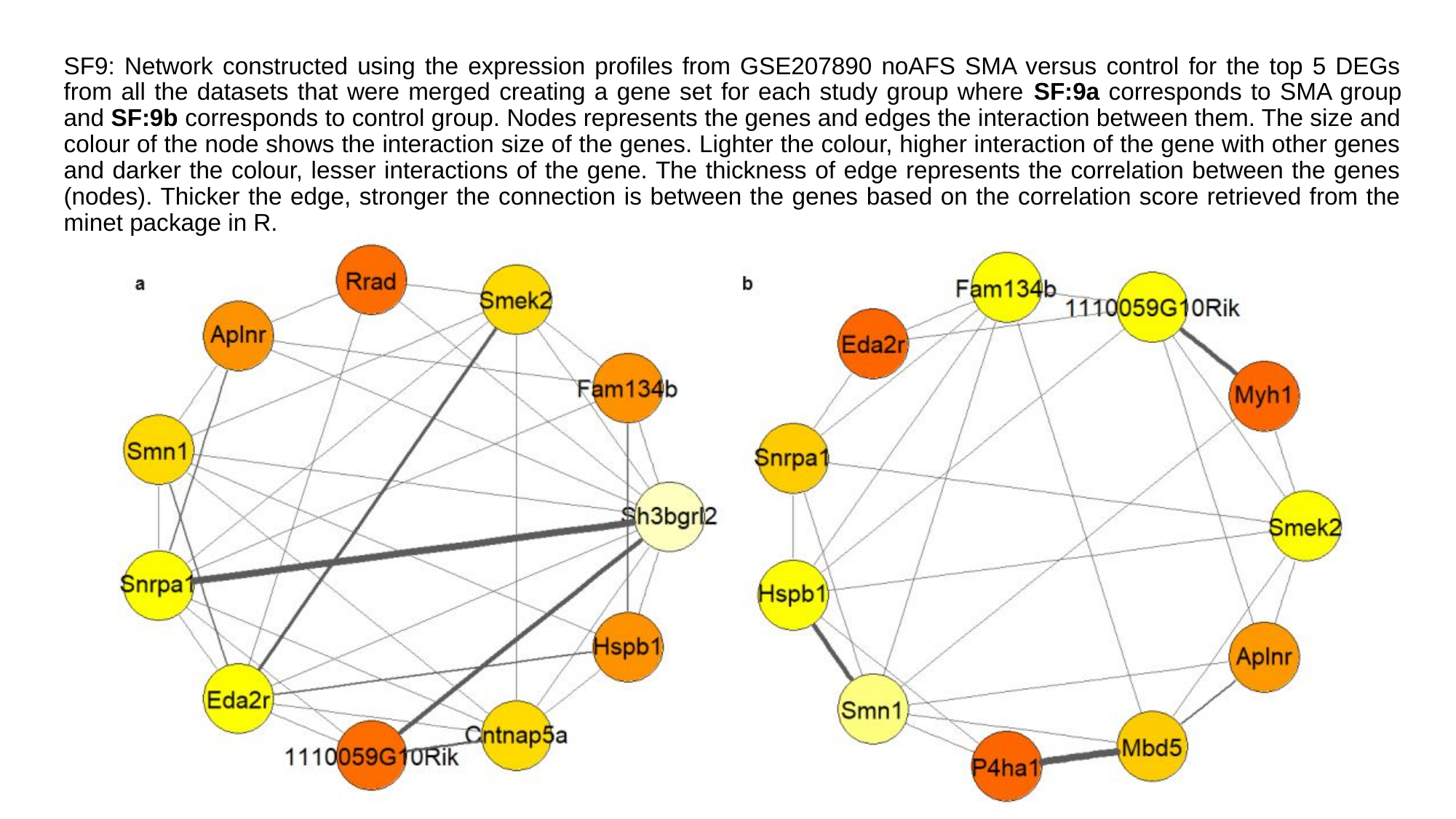

SF9: Network constructed using the expression profiles from GSE207890 noAFS SMA versus control for the top 5 DEGs from all the datasets that were merged creating a gene set for each study group where SF:9a corresponds to SMA group and SF:9b corresponds to control group. Nodes represents the genes and edges the interaction between them. The size and colour of the node shows the interaction size of the genes. Lighter the colour, higher interaction of the gene with other genes and darker the colour, lesser interactions of the gene. The thickness of edge represents the correlation between the genes (nodes). Thicker the edge, stronger the connection is between the genes based on the correlation score retrieved from the minet package in R.

## Slide 14
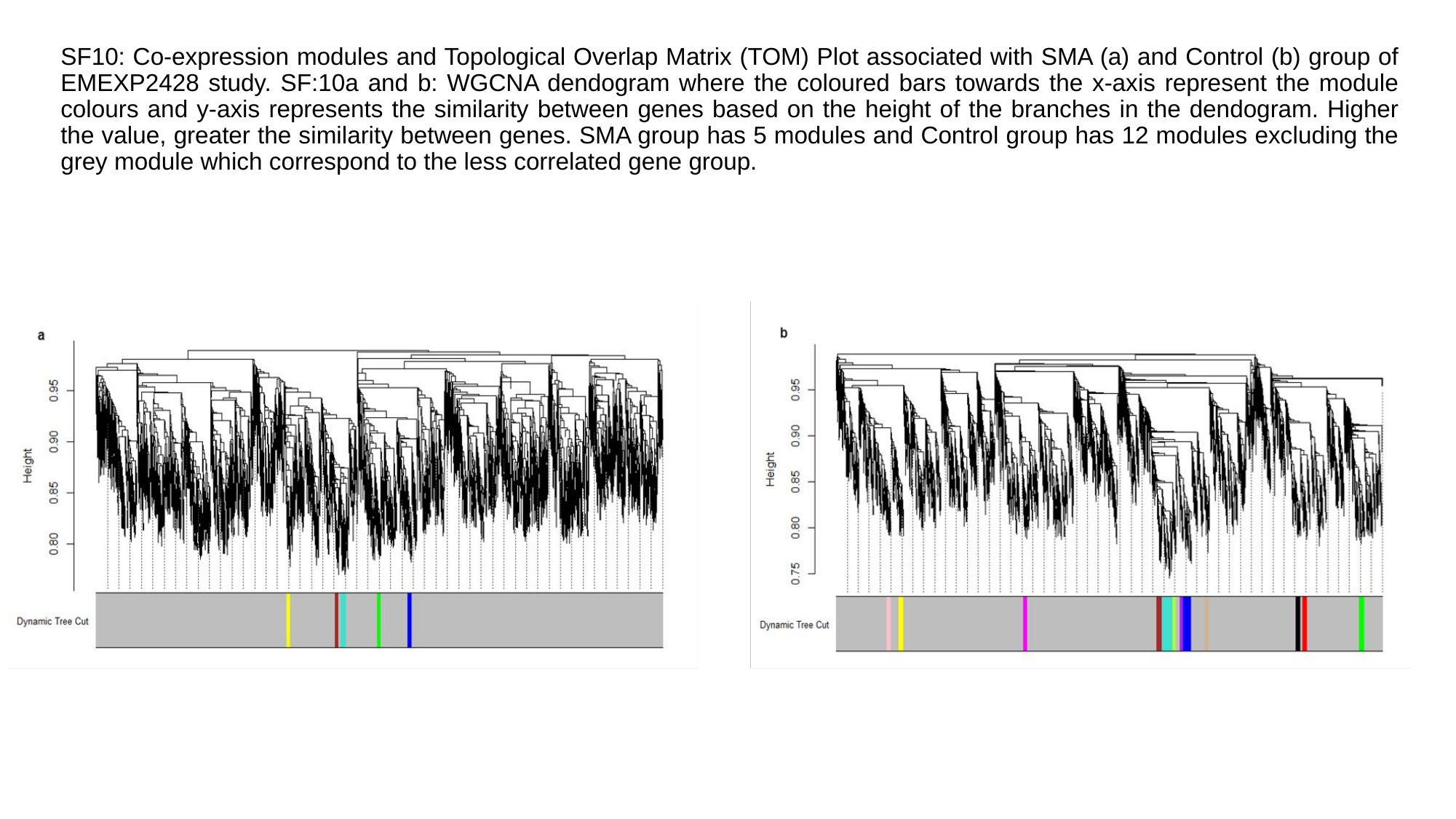

SF10: Co-expression modules and Topological Overlap Matrix (TOM) Plot associated with SMA (a) and Control (b) group of EMEXP2428 study. SF:10a and b: WGCNA dendogram where the coloured bars towards the x-axis represent the module colours and y-axis represents the similarity between genes based on the height of the branches in the dendogram. Higher the value, greater the similarity between genes. SMA group has 5 modules and Control group has 12 modules excluding the grey module which correspond to the less correlated gene group.

## Slide 15
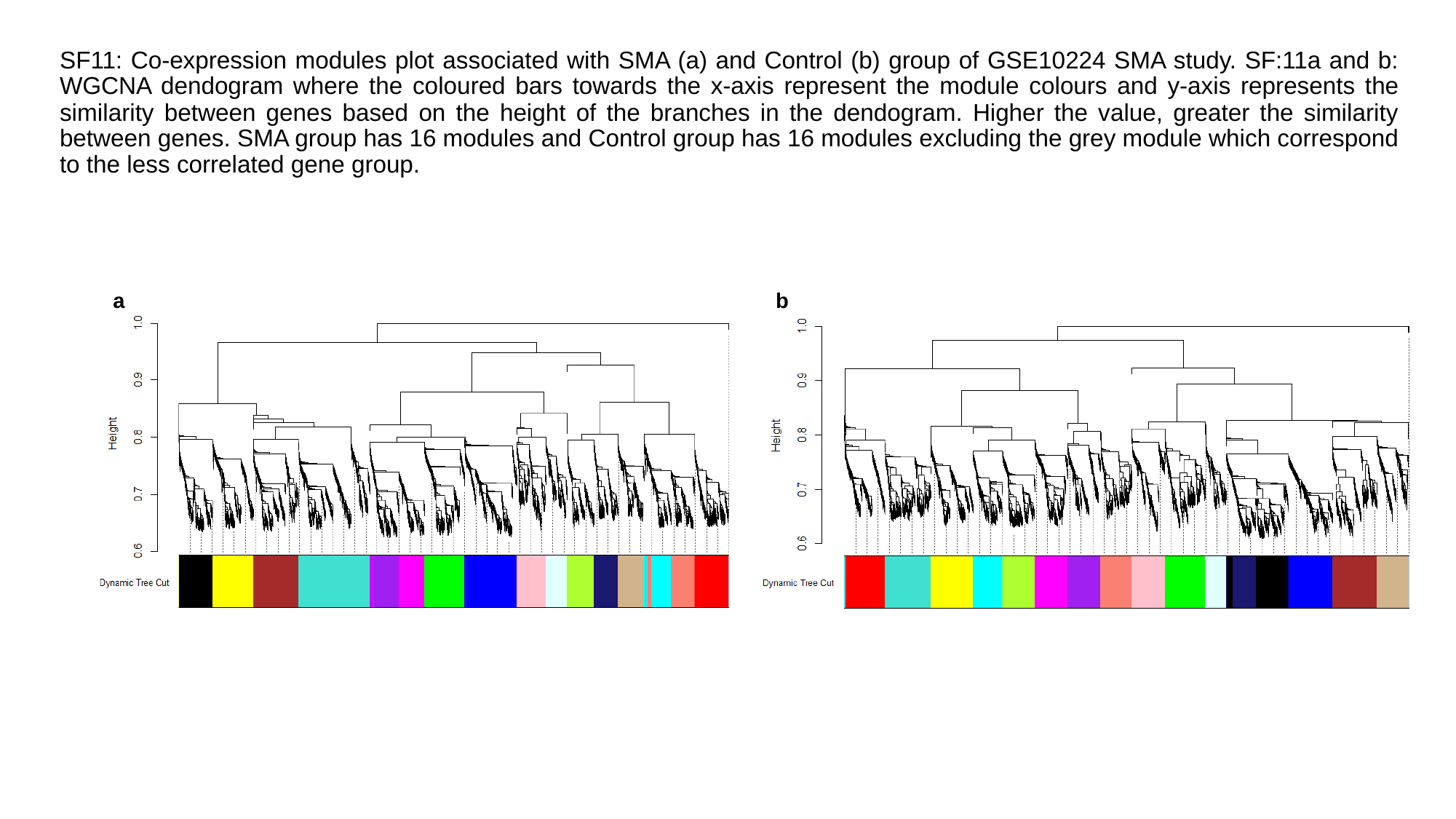

SF11: Co-expression modules plot associated with SMA (a) and Control (b) group of GSE10224 SMA study. SF:11a and b: WGCNA dendogram where the coloured bars towards the x-axis represent the module colours and y-axis represents the similarity between genes based on the height of the branches in the dendogram. Higher the value, greater the similarity between genes. SMA group has 16 modules and Control group has 16 modules excluding the grey module which correspond to the less correlated gene group.
a
b

## Slide 16
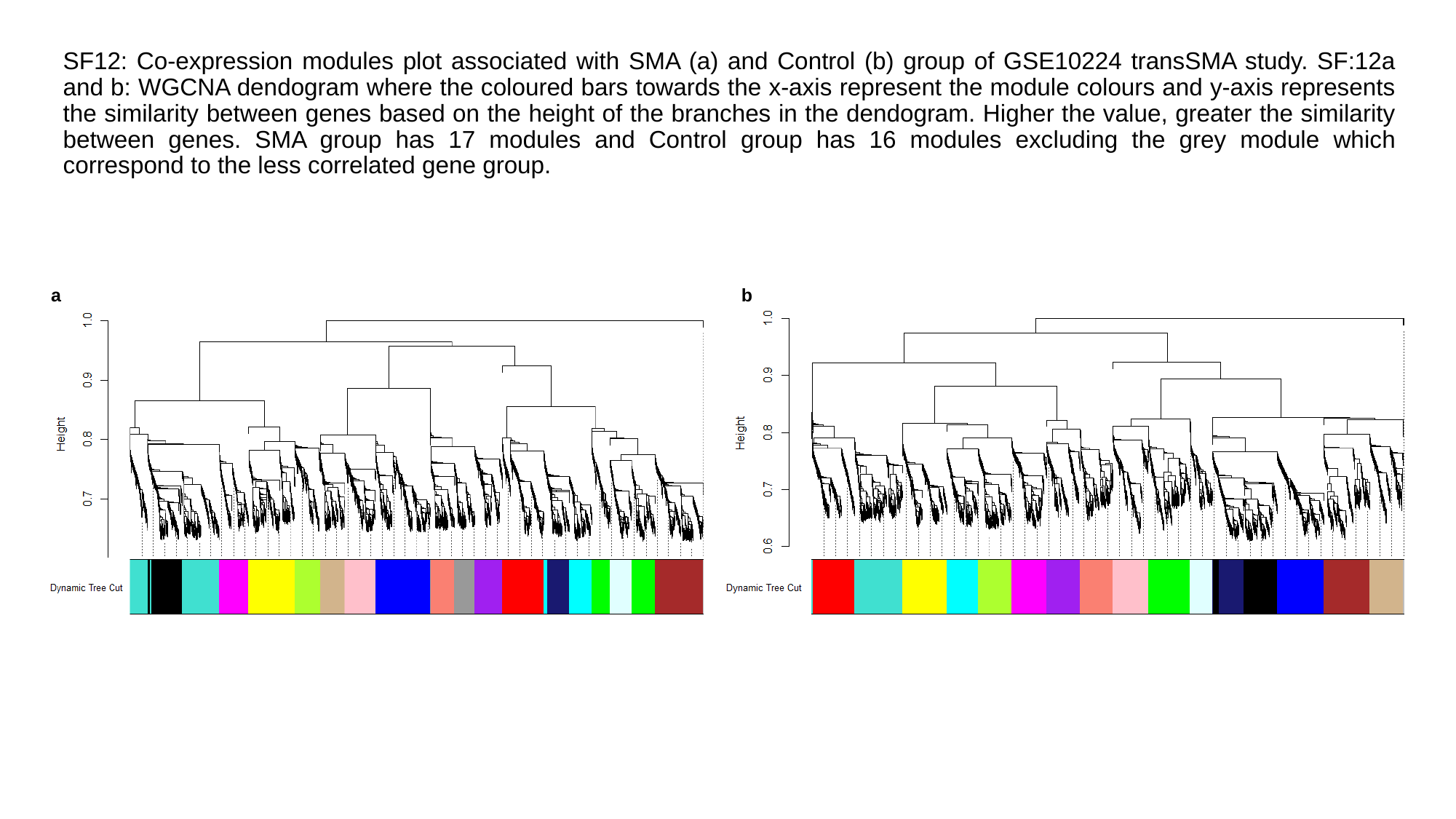

SF12: Co-expression modules plot associated with SMA (a) and Control (b) group of GSE10224 transSMA study. SF:12a and b: WGCNA dendogram where the coloured bars towards the x-axis represent the module colours and y-axis represents the similarity between genes based on the height of the branches in the dendogram. Higher the value, greater the similarity between genes. SMA group has 17 modules and Control group has 16 modules excluding the grey module which correspond to the less correlated gene group.
a
b

## Slide 17
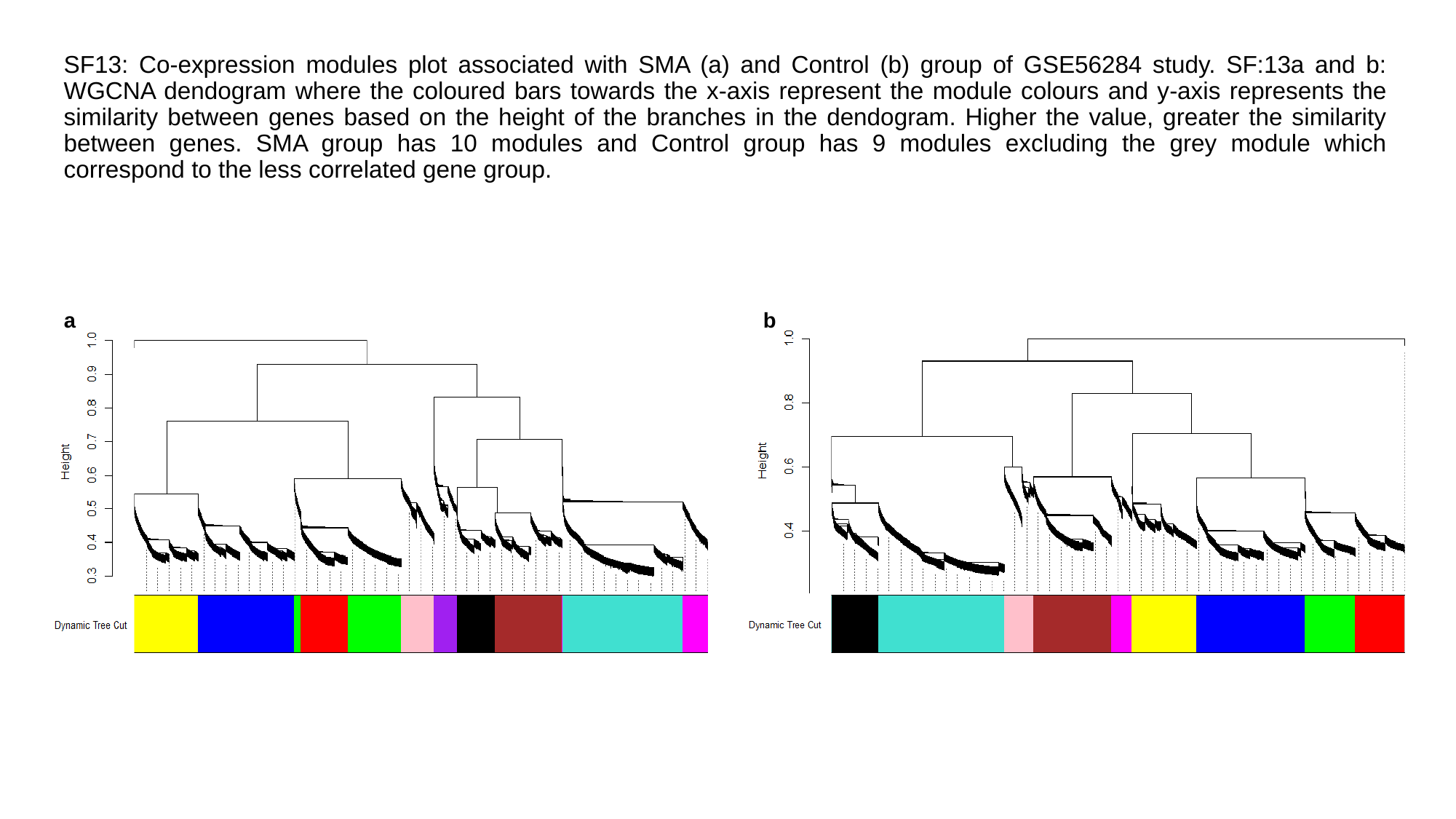

SF13: Co-expression modules plot associated with SMA (a) and Control (b) group of GSE56284 study. SF:13a and b: WGCNA dendogram where the coloured bars towards the x-axis represent the module colours and y-axis represents the similarity between genes based on the height of the branches in the dendogram. Higher the value, greater the similarity between genes. SMA group has 10 modules and Control group has 9 modules excluding the grey module which correspond to the less correlated gene group.
b
a

## Slide 18
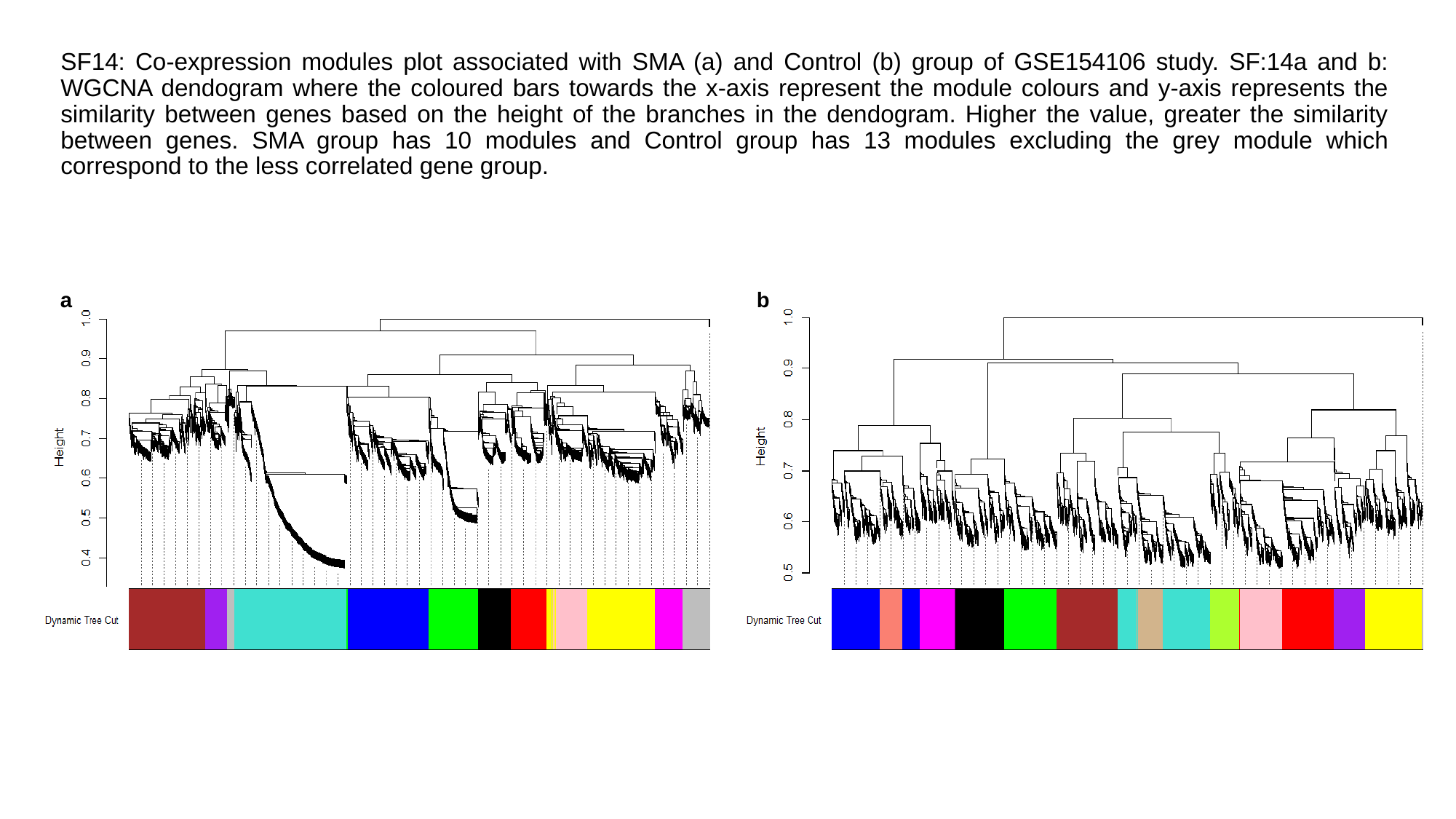

SF14: Co-expression modules plot associated with SMA (a) and Control (b) group of GSE154106 study. SF:14a and b: WGCNA dendogram where the coloured bars towards the x-axis represent the module colours and y-axis represents the similarity between genes based on the height of the branches in the dendogram. Higher the value, greater the similarity between genes. SMA group has 10 modules and Control group has 13 modules excluding the grey module which correspond to the less correlated gene group.
a
b

## Slide 19
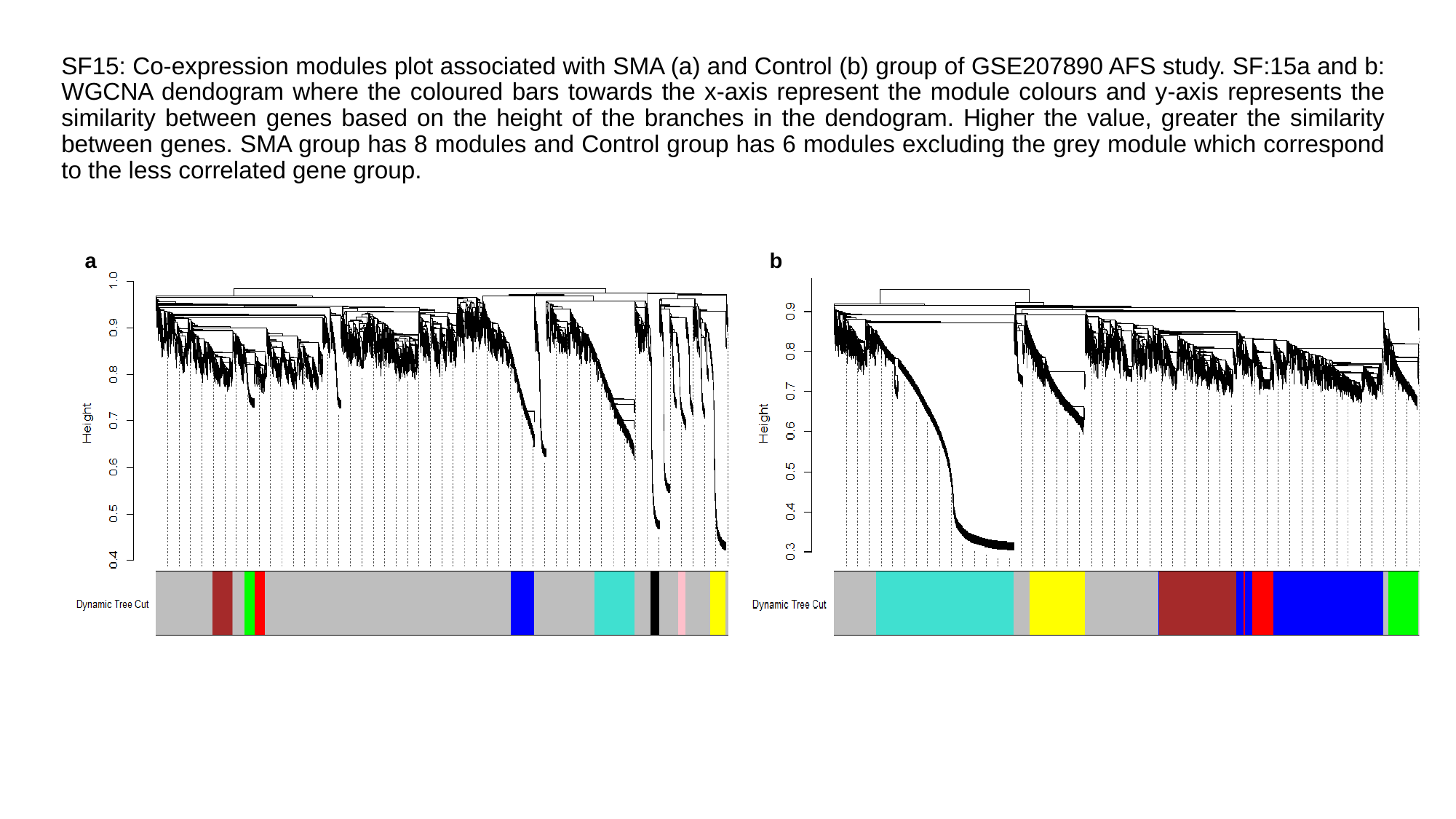

SF15: Co-expression modules plot associated with SMA (a) and Control (b) group of GSE207890 AFS study. SF:15a and b: WGCNA dendogram where the coloured bars towards the x-axis represent the module colours and y-axis represents the similarity between genes based on the height of the branches in the dendogram. Higher the value, greater the similarity between genes. SMA group has 8 modules and Control group has 6 modules excluding the grey module which correspond to the less correlated gene group.
b
a

## Slide 20
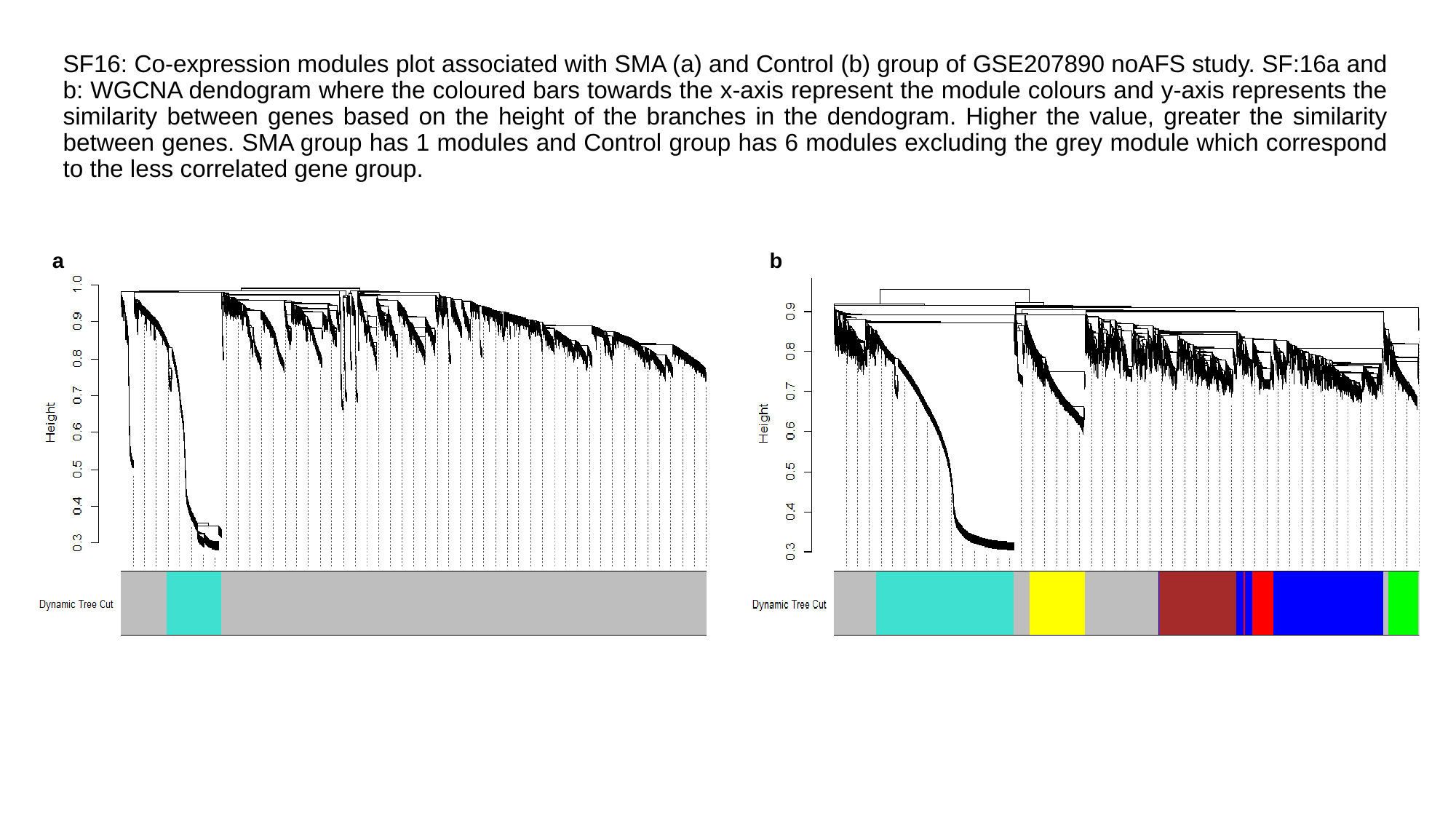

SF16: Co-expression modules plot associated with SMA (a) and Control (b) group of GSE207890 noAFS study. SF:16a and b: WGCNA dendogram where the coloured bars towards the x-axis represent the module colours and y-axis represents the similarity between genes based on the height of the branches in the dendogram. Higher the value, greater the similarity between genes. SMA group has 1 modules and Control group has 6 modules excluding the grey module which correspond to the less correlated gene group.
a
b

## Slide 21
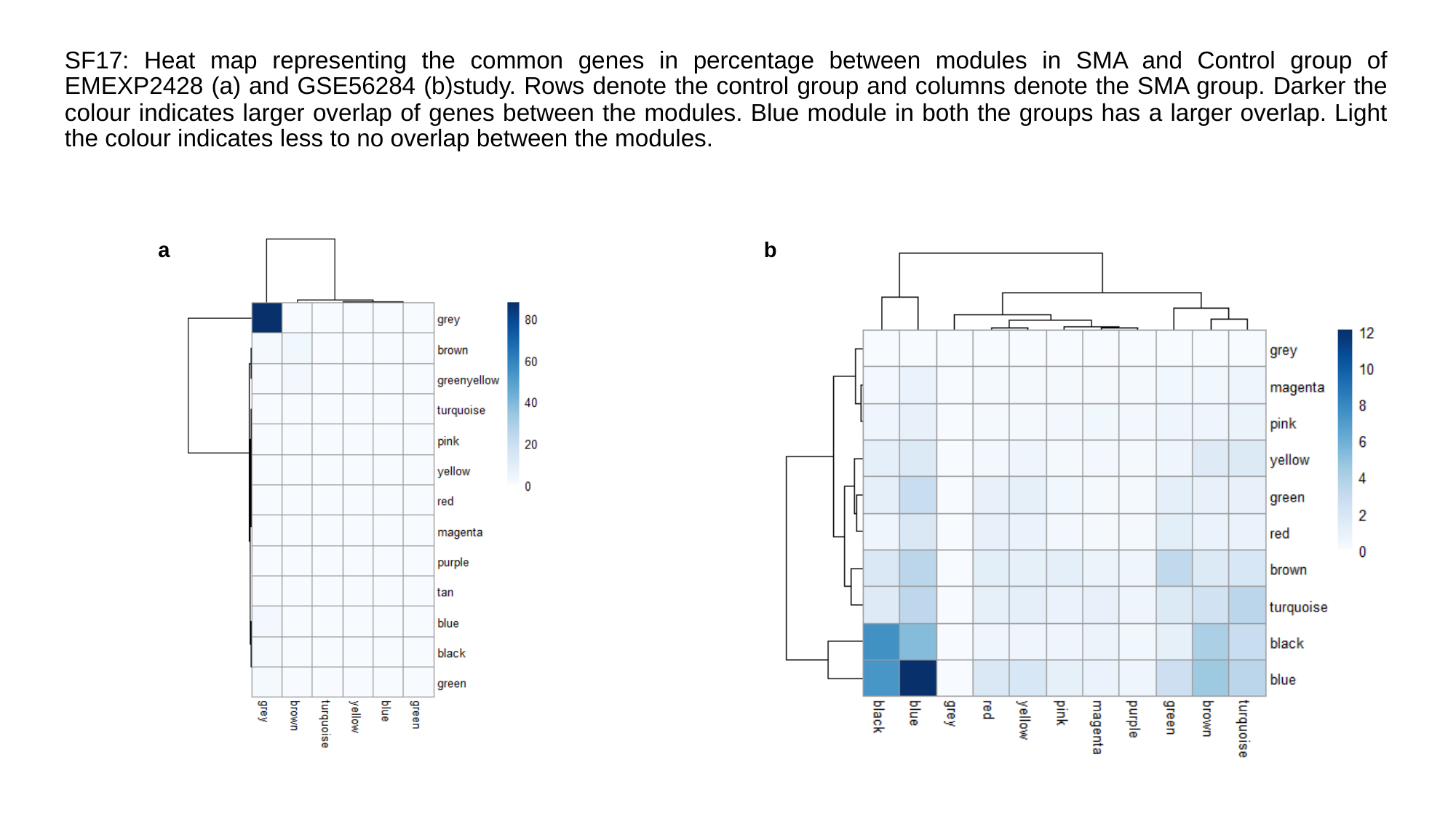

SF17: Heat map representing the common genes in percentage between modules in SMA and Control group of EMEXP2428 (a) and GSE56284 (b)study. Rows denote the control group and columns denote the SMA group. Darker the colour indicates larger overlap of genes between the modules. Blue module in both the groups has a larger overlap. Light the colour indicates less to no overlap between the modules.
a
b

## Slide 22
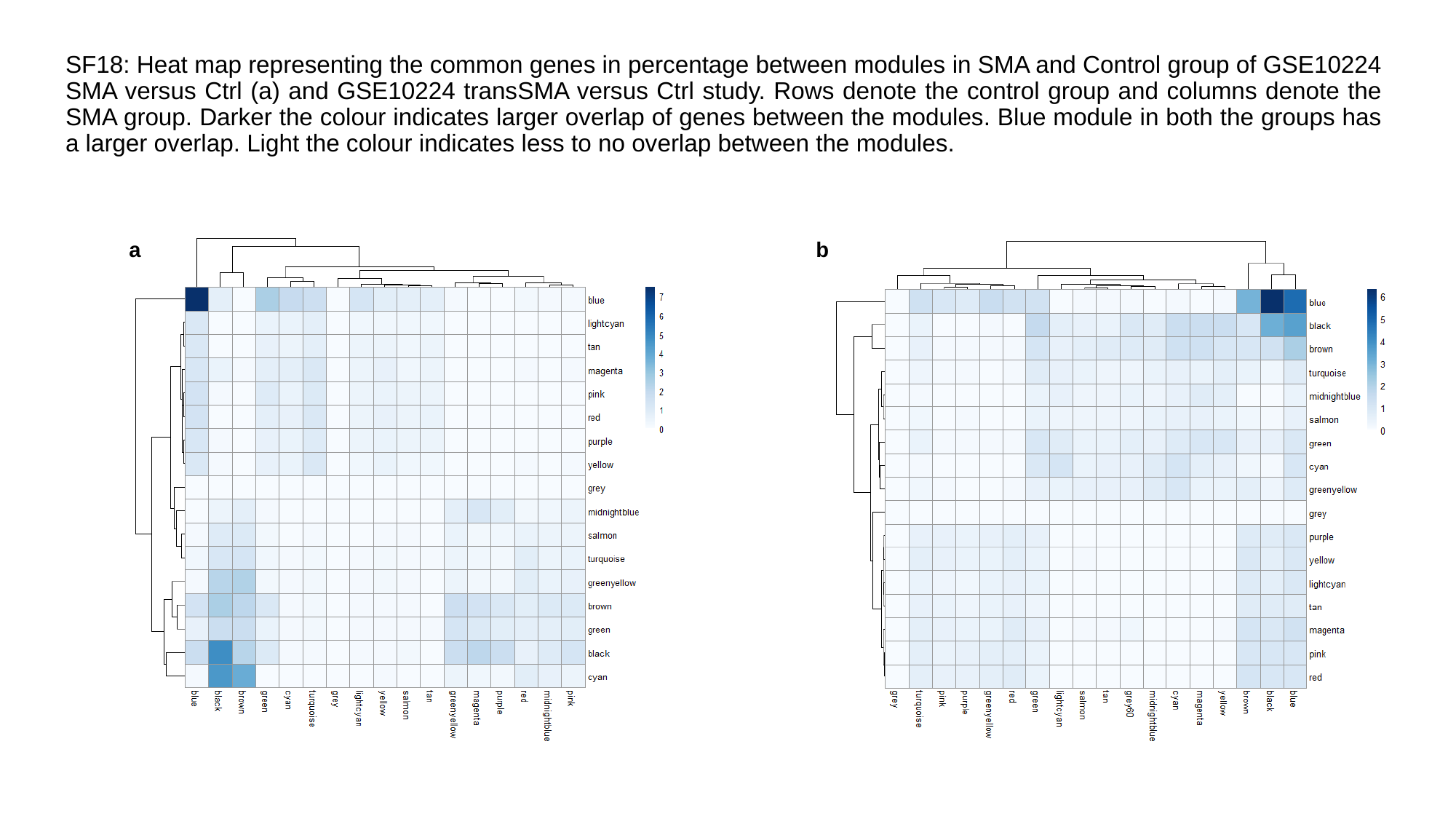

SF18: Heat map representing the common genes in percentage between modules in SMA and Control group of GSE10224 SMA versus Ctrl (a) and GSE10224 transSMA versus Ctrl study. Rows denote the control group and columns denote the SMA group. Darker the colour indicates larger overlap of genes between the modules. Blue module in both the groups has a larger overlap. Light the colour indicates less to no overlap between the modules.
a
b

## Slide 23
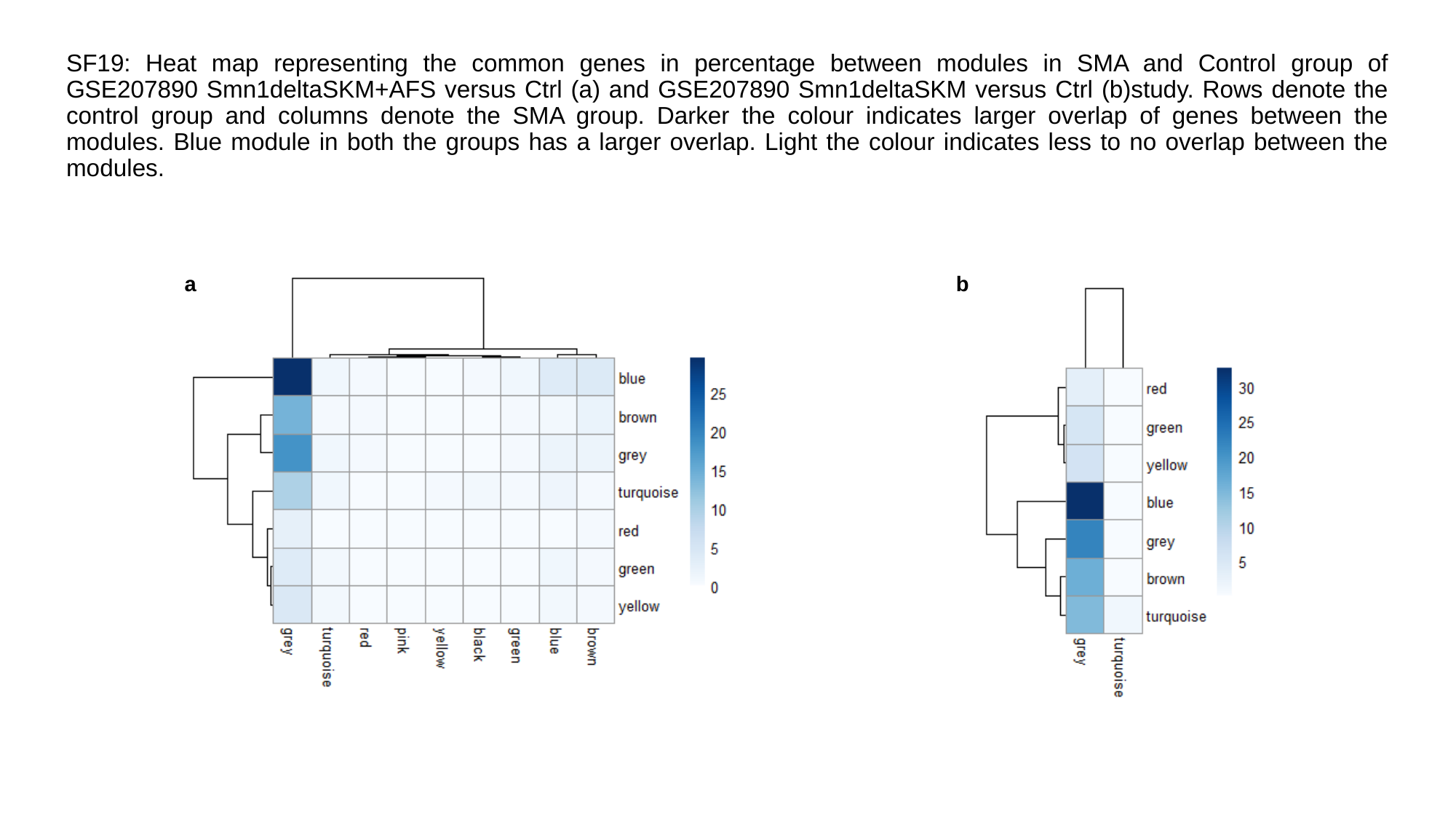

SF19: Heat map representing the common genes in percentage between modules in SMA and Control group of GSE207890 Smn1deltaSKM+AFS versus Ctrl (a) and GSE207890 Smn1deltaSKM versus Ctrl (b)study. Rows denote the control group and columns denote the SMA group. Darker the colour indicates larger overlap of genes between the modules. Blue module in both the groups has a larger overlap. Light the colour indicates less to no overlap between the modules.
b
a

## Slide 24
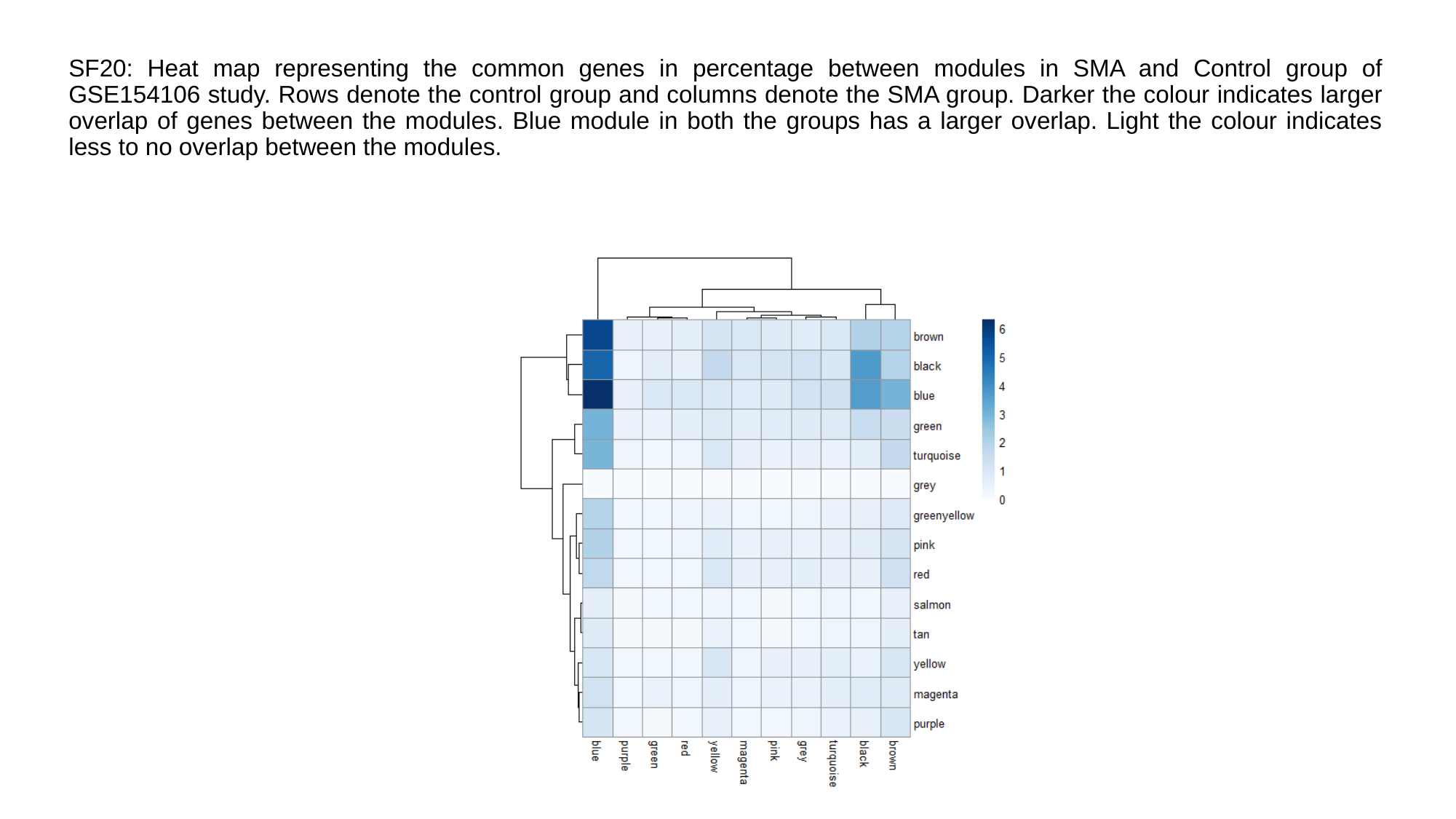

SF20: Heat map representing the common genes in percentage between modules in SMA and Control group of GSE154106 study. Rows denote the control group and columns denote the SMA group. Darker the colour indicates larger overlap of genes between the modules. Blue module in both the groups has a larger overlap. Light the colour indicates less to no overlap between the modules.
