# Supplementary material for: Comparative meta-analysis of transcriptomic studies in spinal muscular atrophy: comparison between tissues and mouse models
Source: BMC Med Genomics. 2024 Nov 12;17:266. doi: 10.1186/s12920-024-02040-0 (PMC11555813; doi:10.1186/s12920-024-02040-0)
Supplement: Supplementary file 2 — Supplementary Material 2. Data S2. WGCNA_SelectionCriterion. [file 12920_2024_2040_MOESM2_ESM.docx]

| **Study Group** | **Soft Threshold Power** | **Minimum Cluster Size** |
| --- | --- | --- |
| GSE207890 Ctrl | 6 | 300 |
| GSE207890 Smn1deltaSKM+AFS | 11 | 600 |
| GSE207890 Smn1deltaSKM | 11 | 600 |
| GSE154106 Ctrl | 7 | 600 |
| GSE154106 SMA | 7 | 600 |
| GSE102204 Ctrl | 7 | 600 |
| GSE102204 SMA | 7 | 600 |
| GSE56284 Ctrl | 9 | 600 |
| GSE56284 SMA | 9 | 600 |
| GSE10224 Ctrl | 10 | 800 |
| GSE10224 SMA | 10 | 800 |
| GSE10224 transSMA | 10 | 800 |
| EMEXP2428 Ctrl | 14 | 100 |
| EMEXP2428 SMA | 14 | 100 |
